# Bimetallic Ca/Zn Nanoagonist Remould the Immunosuppressive Hepatocellular Carcinoma Microenvironment Following Incomplete Microwave Ablation via Pyroptosis and the STING Signaling Pathway

**DOI:** 10.1002/advs.202500670

**Published:** 2025-04-30

**Authors:** Yuan Ling, Xiayi Liang, Kangning Yan, Guichun Zeng, Xiaoqi Zhu, Jinghang Jiang, Shaolong Lu, Xiaobo Wang, Yuying Zhou, Zhaoshen Li, Wei Mai, Duo Wang, Jie Chen

**Affiliations:** ^1^ Department of Hepatobiliary Surgery Department of Medical Ultrasound Department of Traditional Chinese Medicine Guangxi Medical University Cancer Hospital Guangxi Medical University. No. 71 Hedi Road Nanning Guangxi 530021 China; ^2^ Center of Interventional Radiology & Vascular Surgery Department of Radiology Zhongda Hospital Medical School Southeast University Nanjing 210009 China

**Keywords:** cellular pyroptosis, hepatocellular carcinoma, incomplete microwave ablation, JAK2‐STAT3 signaling pathway, STING pathway

## Abstract

During the treatment of solid tumors, local therapeutic approaches carry the risk of incomplete radical cure, which may lead to rapid tumor growth. Incomplete microwave ablation (iMWA) can induce tumors to exhibit highly invasive and uncontrollable growth, which is related to the immunosuppressive microenvironment. A multifunctional bimetallic Ca/Zn nanoagonist (PZH/Zn@CaNA) with a biomimetic liposome‐modified surface to tumor tissues after iMWA is developed. In response to the acidic tumor microenvironment, the released traditional Chinese medicine preparation Pien Tze Huang (PZH) reduced protein expressions of the JAK2‐STAT3 signaling pathway, thereby slowing down the proliferation and growth of hepatocellular carcinoma (HCC). Furthermore, the bimetallic ions Ca^2^⁺ and Zn^2^⁺ can cascade to enhance the killing effect of oxidative stress, generating substantial amounts of reactive oxygen species. This process induces pyroptosis and releases significant quantities of damage associated molecular patterns, thereby triggering immune activation mechanisms related to the STING pathway that reshape the immunosuppressive HCC microenvironment resulting from iMWA. This strategy markedly differs from previous chemoimmunotherapies, which not only effectively addressed the problem of conventional drugs showing heterogeneous distribution in tumor regions, but also verified the critical role played by PZH/Zn@CaNA in inhibiting iMWA‐induced rapid tumor growth, regulating oxidative stress and remodeling the immunosuppressive tumor microenvironment.

## Graphical abstract

The multifunctional bimetallic Ca/Zn nanoagonist (PZH/Zn@CaNA) significantly downregulated the JAK2‐STAT3 signaling pathway, which effectively inhibited the proliferation and growth of HCC with incomplete microwave ablation. Furthermore, the released bimetallic ions (Ca^2^⁺ and Zn^2^⁺) synergistically enhanced oxidative stress‐mediated cytotoxicity, leading to mitochondrial damage and high generation of ROS, which induced tumor cell pyroptosis, release of numerous damage‐associated molecular patterns and remodels the immunosuppressive HCC microenvironment.

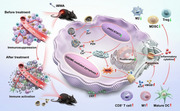

## 1 Introduction

Hepatocellular carcinoma (HCC) is the sixth most common malignant tumor and the third leading cause of death from tumors in the world, and its morbidity and mortality are extremely high.^[^ ^1^ ^]^ Microwave ablation (MWA) is a pivotal strategy for the local treatment of HCC, capable of increasing the 3‐year overall survival rate of HCC patients to 58.7% and significantly enhancing clinical prognosis.^[^ ^2^, ^3^, ^4^, ^5^, ^6^ ^]^ However, MWA carries the risk of incomplete microwave ablation (iMWA), which may have an impact on treatment outcomes.^[^ ^3^, ^7^, ^8^, ^9^, ^10^ ^]^ In areas affected by iMWA, tumor cells may withstand thermal damage and survive, leading to the development of tolerance against thermal ablation treatments.^[^ ^11^, ^12^, ^13^ ^]^ Factors such as hypoxic microenvironments, epithelial‐mesenchymal transition, and angiogenesis that arise during iMWA can accelerate tumor progression.^[^ ^14^, ^15^, ^16^, ^17^ ^]^ Moreover, compared to the transient inflammatory response elicited by complete thermal ablation, the inflammatory response in patients experiencing iMWA tends to be more persistent and complex. This prolonged inflammatory response not only activates anti‐tumor immune mechanisms within residual tumor tissue but also induces an immunosuppressive state that further facilitates rapid growth of residual HCC following iMWA.^[^ ^4^, ^18^, ^19^, ^20^, ^21^, ^22^ ^]^ Consequently, this results in a poor prognosis for HCC patients.

The explosive growth of residual HCC after iMWA is closely related to the immunosuppressive tumor microenvironment.^[^ ^11^, ^15^, ^23^, ^24^ ^]^ The tumor microenvironment (TME) represents a complex and dynamically evolving system wherein circulating immune cells migrate to the tumor site,^[^ ^23^ ^]^ guided by chemokines produced by tumor cells, immune cells, and supportive stromal cells.^[^ ^25^, ^26^, ^27^, ^28^, ^29^ ^]^ The composition of immune components within the TME varies according to the stage of tumor progression iMWA not only accelerates the growth and proliferative properties of residual HCC cells, but also rapidly adapts the TME into a “soil” that promotes the rapid growth of residual cancer “seeds” and evades immune attack.^[^ ^29^, ^30^, ^31^, ^32^ ^]^ The TME of residual tumors after iMWA shows increased accumulation of myeloid‐derived suppressor cells (MDSCs) and decreased expression of cytotoxic T lymphocytes (CTLs), a state of immunodeficiency and immunosuppression that facilitates the invasion and metastasis of HCC.^[^ ^11^, ^24^, ^33^, ^34^, ^35^, ^36^, ^37^, ^38^ ^]^

Pyroptosis is a form of programmed cell death mediated by the Gasdermin protein family (GSDM), characterized by inflammatory necrosis and the release of numerous cytokines.^[^ ^39^, ^40^, ^41^, ^42^ ^]^ This process not only inhibits tumor cell growth but also enhances the anti‐tumor immune response through the recruitment of CD8⁺ T cells and natural killer cells. During pyroptosis, double‐stranded DNA (dsDNA) interacts with metal ions, to activate the cGAS‐STING pathway.^[^ ^43^, ^44^ ^]^ This activation induces the release of type I interferons (IFN), promotes dendritic cell (DC) maturation, and stimulates IFN secretion, significantly enhancing the efficacy of anti‐tumor immunotherapy. The STING pathway serves as a core signaling mechanism within the innate immune system and exhibits a close interaction with pyroptosis, jointly regulating anti‐tumor immunotherapy processes. Upon activation of the STING signaling pathway, there is an enhancement in inflammasome formation alongside upregulation of IFN and other cytokines; this regulation influences both pyroptosis development and innate immune responses.^[^ ^44^, ^45^ ^]^ The effective immune stimulation initiated by pyroptosis has potential to reshape immunosuppressive microenvironments that lack adequate immune responses in residual tumors following iMWA. Furthermore, multiple studies have indicated that activation of the JAK2‐STAT3 signaling pathway can promote HCC occurrence and progression. Inhibition of this pathway may activate GSDM to target cellular membranes for perforation, disrupt osmotic balance within cells leading to swelling and plasma membrane blebbing, ultimately resulting in inflammatory necrosis among tumor cells.^[^ ^45^, ^46^, ^47^, ^48^, ^49^ ^]^ The substantial release of inflammatory factors during this process can enhance antigen availability while triggering robust immune responses.^[^ ^24^, ^50^ ^]^ To tackle the challenges posed by rapid tumor growth and the immunosuppressive microenvironment in iMWA, the combined approach of inhibiting the JAK2‐STAT3 signaling pathway, activating the STING pathway, inducing pyroptosis and oxidative stress in the treatment of iMWA is expected to effectively reshape the immunosuppressive microenvironment lacking immune responses in iMWA residual tumors.

**Scheme 1:**
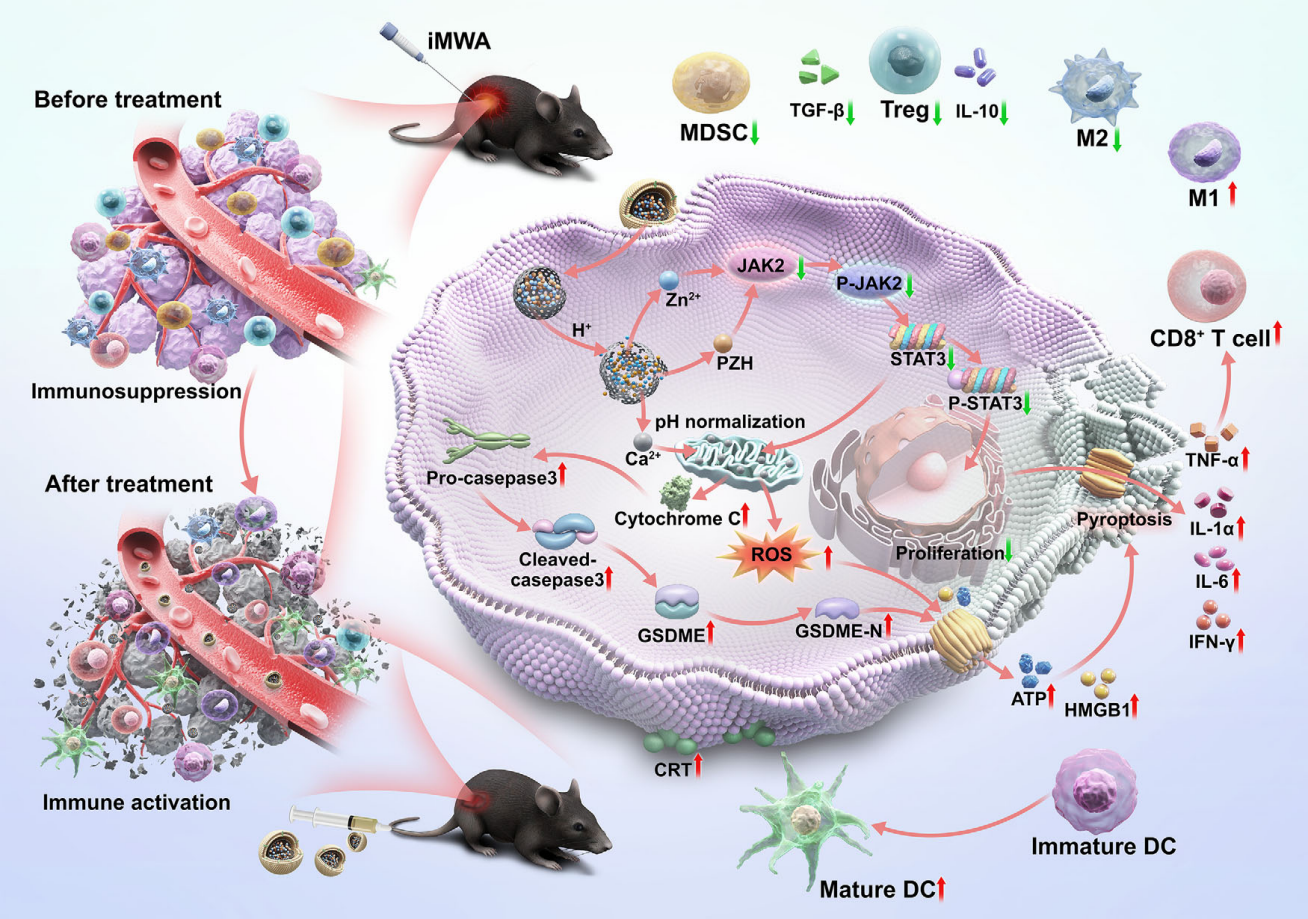
Multifunctional bimetallic Ca/Zn nanoantagonist (PZH/Zn@CaNA) remodels the incomplete microwave ablation tumor microenvironment by inhibiting the activation cascade of the JAK2‐STAT3 pathway in parallel to enhance oxidative stress‐induced pyroptosis in cancer cells. (M1: M1‐like macrophages; M2: M2‐like macrophages; DC: dendritic cells; MDSC: myeloid‐derived suppressor cells; Treg cells: regulatory T cells; CD8: CD8^+^ T cells; CD4: CD4^+^ T cells.).

In this study, based on the fact that the JAK2‐STAT3 signaling pathway can promote the progression of HCC, we observed that the residual tumors after iMWA showed rapid growth and an immunosuppressive state, with high expression levels of JAK‐STAT3‐related proteins. Many studies have emphasized that inhibiting tumor growth, activating immune killing effects, and reshaping the immunosuppressive microenvironment of tumors play a crucial role in controlling the progression and recurrence of residual tumors. Based on the foregoing analysis,^[^ ^4^, ^11^, ^21^, ^34^ ^]^ we innovatively synthesized a biomimetic liposome‐encapsulated bimetallic Ca/Zn nanoagonist (PZH/Zn@CaNA) with a biomimetic membrane phospholipid bilayer structure as a surface modifier to assist PZH/Zn@CaNA to stabilize in the somatic circulation and to enrich into the residual HCC regions, which are highly specific to the liver after iMWA. In response to weakly acidic TME, PZH/Zn@CaNA releases Pien Tze Huang (PZH), Ca^2^⁺, and Zn^2^⁺. PZH not only protects normal hepatocytes,^[^ ^51^, ^52^, ^53^, ^54^ ^]^ but also inhibits tumor cell value‐addition and growth by suppressing the JAK2‐STAT3 signaling pathway, thereby inducing programmed cell death in tumor cells.^[^ ^54^, ^55^, ^56^ ^]^ Thus, PZH, released by PZH/Zn@CaNA in response to the weakly acidic microenvironment of tumors, is able to effectively block the rapid proliferation of cancer cells and induce their death by inhibiting the JAK2‐STAT3 signaling pathway in iMWA residual tumors.^[^ ^57^, ^58^ ^]^ Meanwhile, the released bimetallic Ca^2^⁺ and Zn^2^⁺ not only alleviate the weakly acidic microenvironment of tumors, but also generate a large amount of reactive oxygen species (ROS) through calcium overload and hydrogen peroxide (H₂O₂) depletion, which further disrupts the mitochondrial membrane potential, and promotes the release of mitochondrial DNA as well as the expression of GSDM‐related proteins. In addition, Zn^2^⁺ enhances the recognition of tumor dsDNA by cGAS, which activates the STING signaling pathway and recruits immune cells to the tumor region, thereby remodeling the immunosuppressive microenvironment of iMWA residual tumors.^[^ ^59^, ^60^ ^]^ The novel bimetallic Ca/Zn nanoagonists proposed in this study have the potential to enhance cellular inflammatory programmed necrosis of residual tumor cells following iMWA (**Scheme** **1**). This enhancement is achieved through a multifaceted approach that includes remodeling the immunosuppressive tumor microenvironment, creating a weakly acidic microenvironment, modulating oxidative stress damage effects, inhibiting the JAK2‐STAT3 signaling pathway, and enhancing the STING pathway. These strategies collectively address the limitations posed by immune insufficiency and an immunosuppressive microenvironment on chemoimmunotherapy for HCC, thereby paving the way for future advancements in HCC treatment.

## 2 Results and Discussion

### 2.1 Incomplete Microwave Ablation of HCC Drives Accelerated Local Tumor Growth by Immunosuppression

**Figure 1:**
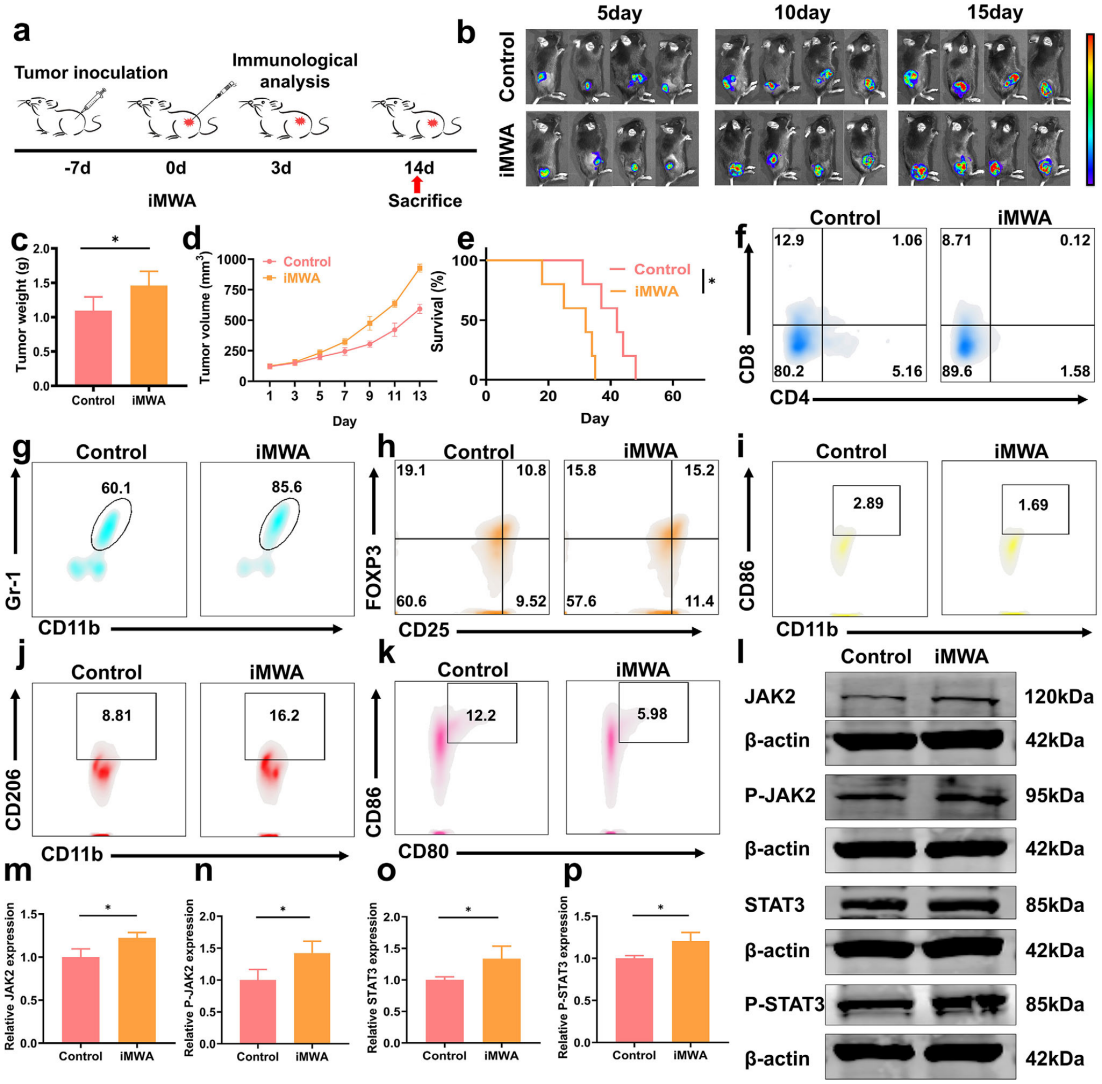
Effects of iMWA on HCC progression and immunity. a) Schematic diagram of the iMWA mechanism used in mice. b) Biofluorescence imaging images of residual tumors after different treatments in C57BL/6J mice. c) Residual tumor weight statistics after different treatments. d) Growth curves of tumors after different treatments. e) Survival curves of mice after different treatments. f) Flow cytometry analysis of CD8^+^ T cells /CD4^+^ T cells, g) MDSC cells, h) Treg cells, i) M1‐like macrophages, j) M2‐like macrophages, k) DC cells. l) WB analysis of JAK2‐STAT3 signaling pathway related proteins in Hepa1‐6 cells after different treatments. m) Quantitative analysis of WB images of JAK2, n) P‐JAK2, o) STAT3, p) P‐STAT3. (*p < 0.05, **p < 0.01, ***p < 0.001, ****p < 0.0001).

To assess the alterations in local tumor growth and tumor immune microenvironment in hepatocellular carcinoma after iMWA,^[^ ^11^, ^13^, ^21^ ^]^ this study involved the treatment of iMWA HCC mice (**Figure** **1**a). Our findings revealed that local tumors in mice subjected to iMWA exhibited a phase of explosive growth when compared to those in the untreated group (Figure 1b), while residual tumor tissues post‐iMWA demonstrated rapid proliferation (Figure 1b). At the designated cut‐off time for treatment, both the volume and weight of residual tumors were significantly greater than those observed in untreated hormonal mice (Figure 1c,d). We also monitored the survival rates of the different treatment groups on a 70‐day cycle, and the survival curves showed that tumor‐bearing mice in the iMWA group had lower survival rates compared to the control group (Figure 1e). This suggests a multiplicative growth rate within the residual tumors after iMWA, potentially accompanied by specific mutations leading to thermal ablation tolerance, drug resistance, immunosuppression, and decreased survival rates.

To further investigate the immunoediting of the HCC tumor microenvironment triggered by iMWA, we collected residual HCC tumor tissues three days post‐iMWA and conducted flow cytometry (FCM) to analyze changes in immunophenotypes within the TME. The results from FCM analysis confirmed that, compared to untreated HCC tissues, residual HCC tissues following iMWA exhibited a significantly elevated expression of CD11b^+^Gr‐1^+^ MDSCs (Figure 1g; Figure S1a, Supporting Information). Additionally, regulatory T cells (Tregs), a subpopulation of T cells known for their potent immunosuppressive effects, showed an increased presence of CD25^+^FOXP3^+^ Treg cells in the residual tumors after iMWA as indicated by FCM analysis results (Figure 1h; Figure S1f, Supporting Information), which can inhibit immune responses from other cell types. Subsequently, we observed a trend indicating decreased levels of tumor‐associated macrophages (TAMs) with respect to M1/M2 polarization (Figure S1b–d, Supporting Information). Specifically, M1‐type macrophages characterized as CD11b^+^CD86^+^ TAMs‐M1 were found at reduced levels within the expression levels in this environment (Figure 1i; Figure S1b, Supporting Information). Furthermore, there was a decline in both CD80^+^CD86^+^ dendritic cells (DCs)—the most efficient antigen presenters within the immune system—and CD3^+^CD8^+^ cytotoxic T lymphocytes (CTLs) following iMWA treatment as shown in Figure 1f,k respectively. The observed decrease in expression levels among these immune cell populations suggests an overall immunosuppressive state within the TME of residual tumors post‐iMWA. This condition is characterized by an increased accumulation of myeloid‐derived suppressor and regulatory T cells alongside diminished infiltration of CTLs, M1/M2 macrophages, and DCs. These findings imply that remodeling the immunosuppressive microenvironment surrounding tumors plays a crucial role in their progression.

The JAK2‐STAT3 signaling pathway is a crucial intracellular signaling cascade that plays a significant role in cell growth, differentiation, proliferation, and apoptosis. Several studies have indicated that JAK2 proteins and STAT3 proteins are overexpressed in HCC cells.^[^ ^61^ ^]^ Notably, the expression levels of STAT3 proteins correlate with the prognosis of HCC patients, suggesting their involvement in HCC development. Furthermore, the phosphorylation of JAK2 and STAT3 has been shown to facilitate the invasion and metastasis of HCC cells without affecting cellular growth or proliferation.^[^ ^62^ ^]^ To investigate the mechanisms underlying rapid tumor regrowth following iMWA, we assessed the expression levels of proteins associated with the JAK2‐STAT3 signaling pathway in residual tumors post‐iMWA. Immunofluorescence detection along with quantitative analysis was conducted for JAK2, STAT3, P‐JAK2, and P‐STAT3 (Figures S2a–e). The results demonstrated an upregulation of both JAK2 and STAT3 as well as their phosphorylated forms after iMWA, which were corroborated by Western blot (WB) bands and grayscale quantitative analysis (Figure 1l–p). This suggests that the accelerated growth observed in residual tumors following iMWA is linked to activation of the JAK2‐STAT3 signaling pathway. The combined activation of this pathway within residual HCC tumors post‐iMWA may be attributed to immunosuppression and immune exhaustion phenomena which ultimately drive enhanced tumor progression. Therefore, addressing immune dysregulation while inhibiting pathways responsible for tumor growth holds significant therapeutic potential against uncontrolled tumor expansion.

### 2.2 Synthesis and Characterization of Bimetallic Ca/Zn Nanoagonist

**Figure 2:**
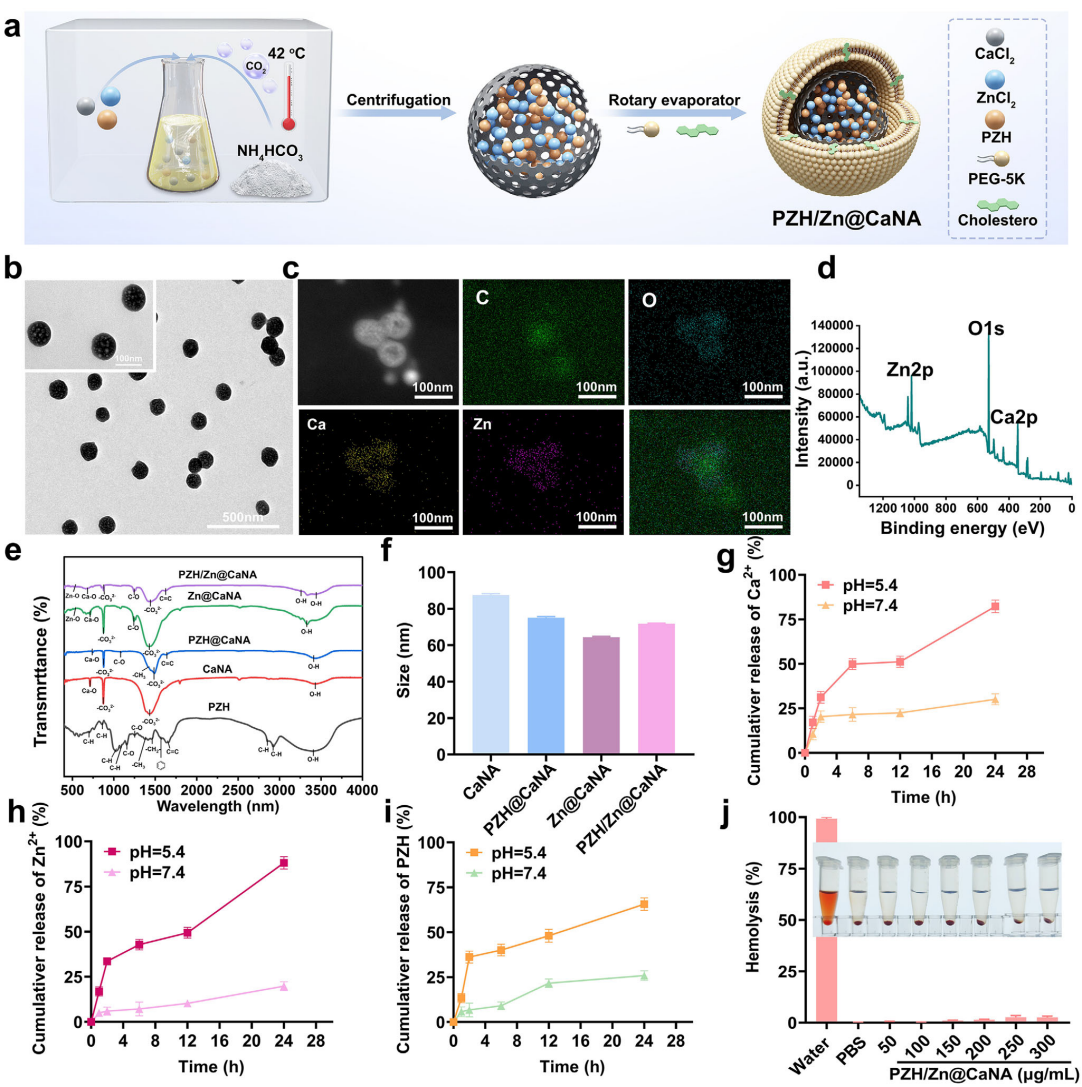
Synthesis and characterization of the bimetallic Ca/Zn nanoagonist PZH/Zn@CaNA. a) Schematic representation of the synthesis process of PZH/Zn@CaNA. b) TEM images of the PZH/Zn@CaNA (scar bar: 500, 100 nm). c) Elemental mapping of C, O, Ca, Zn of PZH/Zn@CaNA (scar bar: 100 nm). d) XPS of PZH/Zn@CaNA. e) FTIR spectrum of PZH/Zn@CaNA. f) Particle sizes of CaNA, PZH@CaNA, Zn@CaNA and PZH/Zn@CaNA. g) Drug release profiles of Ca^2+^, h) Zn^2+^ and i) PZH at pH 5.4 and 7.4. j) Hemolysis assay of blood cells at different concentrations of PZH/Zn@CaNA.

We synthesized the Bimetallic Ca/Zn Nanoagonist by employing a modified “one‐pot gas” method.^[^ ^14^, ^63^ ^]^ In this approach, anhydrous ethanol containing CaCl_2_, ZnCl_2_, and PZH were placed in an airtight container. Carbon dioxide (CO_2_) was continuously generated from ammonium bicarbonate (NH_4_HCO_3_) at the bottom of the container at 42 °C and delivered to the mixture as a gas flow. This process facilitated the formation of spherical calcium carbonate encapsulating both PZH and Zn^2^⁺. The continuous gas flow promoted chemical reactions that resulted in spherical calcium carbonate structures encapsulating PZH and Zn^2^⁺. To ascertain the optimal ratio of bimetallic ions, we have fabricated a series of nanocarriers featuring diverse mass ratios of calcium chloride to zinc chloride, and selected the ratio with the most potent ROS generation capacity via ROS probe detection and assessment (Figure S3, Supporting Information). To enhance biosafety, we further surface‐modified these spherical calcium carbonate particles with a liposome structure designed to mimic a phospholipid bilayer, yielding the Bimetallic Ca/Zn Nanoagonist designated as PZH/Zn@CaNA (**Figure** **2**a). Transmission electron microscopy (TEM) images clearly illustrate the spherical morphology of PZH/Zn@CaNA (Figure 2b). Elemental mapping analysis (Figure 2c) and energy‐dispersive X‐ray spectroscopy (EDS) confirmed that both calcium and zinc were uniformly distributed within PZH/Zn@CaNA (Figure S4, Supporting Information). The chemical state of PZH/Zn@CaNA was assessed using X‐ray photoelectron spectroscopy (XPS), which revealed that both Ca^2^⁺ and Zn^2^⁺ existed in their +2 oxidation states (Figure 2d; Figure S5a,b, Supporting Information). Fourier Transform Infrared Spectroscopy (FTIR) was employed to identify the IR absorption peaks of various products generated during the stepwise synthesis process (Figure 2e). The results indicated that the IR absorption spectrum of PZH/Zn@CaNA exhibited peaks analogous to those of three substances: PZH, CaNA, and Zn@CaNA. These included C = C and C‐O stretching and vibrational absorption peaks associated with PZH, as well as stretching and bending vibrational absorption peaks characteristic of carbonate, along with distinct absorption features related to Ca‐O and Zn‐O bonds. This evidence confirms that spherical calcium carbonate effectively encapsulated both PZH and Zn^2+^. Additionally, measurements obtained through dynamic light scattering (DLS) for particle size (Figure 2f) and zeta potential (Figure S6, Supporting Information) did not exhibit significant variations. This observation suggests that the encapsulation of PZH and Zn^2+^ does not substantially influence the particle size characteristics of the nanoparticles.

In order to verify the content of calcium carbonate, nanoparticles encapsulated PZH in the Chinese medicine composite preparation, the LC‐MS/MS analysis indicated that the encapsulation efficiency of PZH was 74.8%, while the drug loading capacity reached 78.7%. Subsequently, to simulate the weakly acidic microenvironment characteristic of tumors and investigate the H^+^‐responsive decomposition of PZH/Zn@CaNA along with subsequent release of Ca^2+^, Zn^2+^, and PZH (Figure 2g–i), we placed 2 ml of a PZH/Zn@CaNA solution (6 mg ml^−1^) inside a dialysis bag immersed in media at pH levels of 7.4 and 5.4. At various time points, 3 mL samples were extracted from the peripheral medium, and the release percentage and concentration of the drug were analyzed by means of inductively coupled plasma (ICP) spectroscopy (Figure 2i) and liquid chromatography – tandem mass spectrometry (LC‐MS/MS) (Figure S7, Supporting Information). The findings revealed that after 24 h at pH 5.4, there was an approximate release rate of Ca^2+^ reaching up to 80% and Zn^2+^ approximately at 85%, compared to their respective release rates under neutral pH conditions. Additionally, the release rate for PZH attained ≈65%, which is double that observed at pH level 7.4 (Figure 2i). This suggests that PZH/Zn@CaNA exhibits favorable acid‐responsive release characteristics potentially beneficial for enhancing tumor microenvironments. Moreover, stability assessments demonstrated that PZH/Zn@CaNA maintained a high degree of stability across various media including PBS, anhydrous ethanol, DMEM, and fetal bovine serum (FBS). Digital photographs indicated no significant aggregation or notable changes in particle size for PZH/Zn@CaNA (Figure S8, Supporting Information). Furthermore, hemolysis experiments conducted using extracted mouse blood cells confirmed that varying concentrations of PZH/Zn@CaNA did not induce hemolysis in blood cells (Figure 2j). These results imply that PZH/Zn@CaNA possesses good biocompatibility conducive to further evaluations regarding its performance in vitro as well as validation within vivo biomedical applications.

### 2.3 In Vitro Validation of the Anti‐Tumor Properties of Bimetallic Ca/Zn Nanoagonist Against Hepatocellular Carcinoma Cells

**Figure 3:**
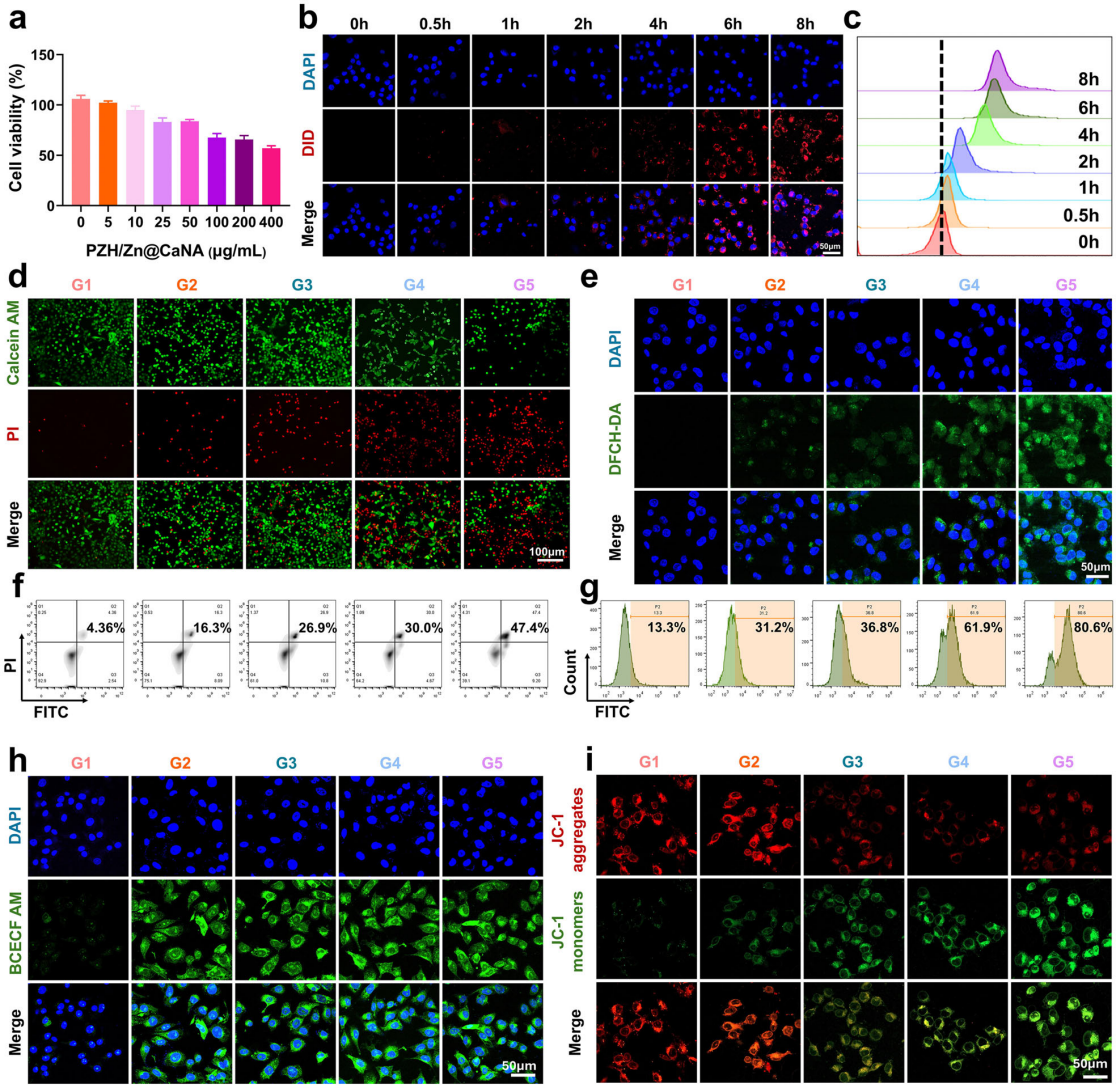
In vitro assessment of PZH/Zn@CaNA targeting and activation of oxidative stress. a) Cell viability of Hepa1‐6 cells after treatment with different concentrations of PZH/Zn@CaNA. b) CLSM images of Hepa1‐6 cells incubated with DID‐labelled PZH/Zn@CaNA for different durations, c) Comparison of phagocytosis fluorescence intensity of Hepa1‐6 cells in response to DID‐labelled PZH/Zn@CaNA incubated for different durations. d) Fluorescence images of Hepa1‐6 cells co‐stained with Calein AM/PI after different treatments (scar bar: 100 µm). e) CLSM images of ROS production in Hepa1‐6 cells after different treatments (scar bar: 50 µm). f) FCM of AnnexinV‐FITC/PI double staining of Hepa1‐6 cells for apoptosis after different treatments. g) FCM of ROS in Hepa1‐6 cells after different treatment groups. h) CLSM image of pH levels of Hepa1‐6 cells after different treatments (scar bar: 50 µm). i) CLSM images of mitochondrial membrane dysfunction in Hepa1‐6 cells assessed using JC‐1 (aggregates in red, monomers in green) after different treatments (scar bar: 50 µm). (G1: Control; G2: CaNA; G3: PZH@CaNA; G4: Zn@CaNA; G5: PZH/Zn@CaNA).

PZH/Zn@CaNA is recognized for its excellent biocompatibility and specific cytotoxic effects on HCC cells. The proliferation and toxicity of PZH/Zn@CaNA on normal hepatocytes and HCC cells were assessed using the Cell Counting Kit‐8 (CCK8). It was observed that high concentrations of PZH/Zn@CaNA did not exhibit significant toxicity toward normal hepatocytes (Figure S9, Supporting Information), while they markedly inhibited the growth of HCC cells (**Figure** **3**a). This effect is believed to be associated with the weakly acidic microenvironment present in the TME of HCC, which facilitates the release of encapsulated PZH and Zn^2+^ through the decomposition of liposomes and calcium carbonate shells. The results from ethane dithiothreitol (EdU) assays evaluating cell proliferative properties at various concentrations of PZH/Zn@CaNA indicated that concentrations exceeding 200 µg/ml significantly inhibited cell proliferation in HCC cells (Figures S10 and S11, Supporting Information). Additionally, PZH/Zn@CaNA was loaded with red DID dye and co‐incubated with Hepa1‐6 cells at different time points. Confocal laser scanning microscopy (CLSM) revealed that by the end of the incubation period at 8 h, Hepa1‐6 cells had internalized a substantial amount of DID‐PZH/Zn@CaNA, exhibiting strong red fluorescence intensity (Figure 3b). These findings were corroborated by FCM analysis demonstrating effective endocytosis of DID‐PZH/Zn@CaNA by HCC cells (Figure 3c; Figure S12, Supporting Information). Collectively, these results suggest that PZH/Zn@CaNA can be effectively internalized and concentrated within Hepa1‐6 cancer cells to exert subsequent cytotoxic effects against them.

A number of studies utilizing calcium carbonate nanoparticles as drug carriers have demonstrated promising prospects and performance, primarily due to their ability to enhance the weakly acidic pH in the TME and release Ca.^2+[^ ^64^, ^65^ ^]^ This release can lead to mitochondrial damage through calcium overload and increased ROS, which subsequently activate cytochrome c (CytC) proteins and Caspase‐3 proteins, resulting in gasdermin E (GSDME) cleavage and inducing cellular pyroptosis.^[^ ^39^, ^66^ ^]^ Based on these findings, we constructed PZH/Zn@CaNA by combining Zn^2+^—which promote cellular pyroptosis and immune response—with PZH, known for its capacity to inhibit the JAK2‐STAT3 signaling pathway. After various treatments (G1: Control; G2: CaNA; G3: PZH@CaNA; G4: Zn@CaNA; G5: PZH/Zn@CaNA), double staining of live and dead cells using Calcein/PI revealed that the PZH/Zn@CaNA group exhibited the highest penetration into the cell interior of hepatocellular carcinoma cells, as indicated by red fluorescence from Propidium Iodide (PI). This was evidenced by an intense red fluorescence signal compared with other groups (Figure 3d). Furthermore, FCM analysis of early apoptosis and late apoptosis rates among HCC cells subjected to different treatments showed that the overall apoptosis rate in the PZH/Zn@CaNA group reached 56.6% (Figure 3f; Figure S13, Supporting Information).

PZH/Zn@CaNA exhibited a highly efficient cytotoxic effect on Hepa1‐6 HCC cells, which was associated with mitochondrial damage and the activation of programmed cell death pathways. The internalization of PZH/Zn@CaNA into the cells resulted in a reaction with intracellular H^+^, leading to the decomposition of calcium carbonate shells and an enhancement of the weakly acidic pH within TME. The changes in pH within Hepa1‐6 cells were monitored using a pH probe, confirming that groups containing calcium carbonate nanoshells exhibited significantly increased pH levels and displayed strong green fluorescence compared to the control group (Figure 3h; Figure S14, Supporting Information). Additionally, Ca^2+^ released from these nanoshells were detected using a Ca^2+^ probe and visualized through CLSM, revealing pronounced green fluorescence indicative of Ca^2+^ presence in groups containing calcium carbonate nanoshells. These findings aligned with results obtained from simulating a weakly acidic tumor microenvironment in vitro (Figure S15a,b, Supporting Information). The release of Ca^2+^ can accumulate within mitochondria, resulting in damage to mitochondrial membrane potential. By employing CLSM and FCM, it was observed that HCC cells in the PZH/Zn@CaNA group exhibited a decrease in mitochondrial membrane potential, accompanied by calcium deposition and ROS production (Figure 3i; Figure S16a–c, Supporting Information). Notably, JC‐1 failed to accumulate within the mitochondrial matrix; instead, it generated green fluorescence indicative of JC‐1 monomers, which subsequently initiated programmed cell death. The combination of Zn^2+^ with Ca^2+^ facilitated electron leakage from mitochondrial aerobic respiration and oxidation of NADPH via NOX1, thereby promoting substantial ROS accumulation within the cells. Consequently, when assessing ROS production levels across different treatment groups using ROS probes, quantitative analyses through CLSM and FCM revealed that the highest ROS levels were present in HCC cells from the PZH/Zn@CaNA group (Figure 3e,g; Figures S17 and S18, Supporting Information). This finding underscores its role in inducing cancer cell cytotoxicity.

### 2.4 In Vitro Investigation of the Mechanisms Underlying the Remodeling of the Immunosuppressive Microenvironment and the Inhibition of Hepatocellular Carcinoma Growth by Bimetallic Ca/Zn Nanoagonist

**Figure 4:**
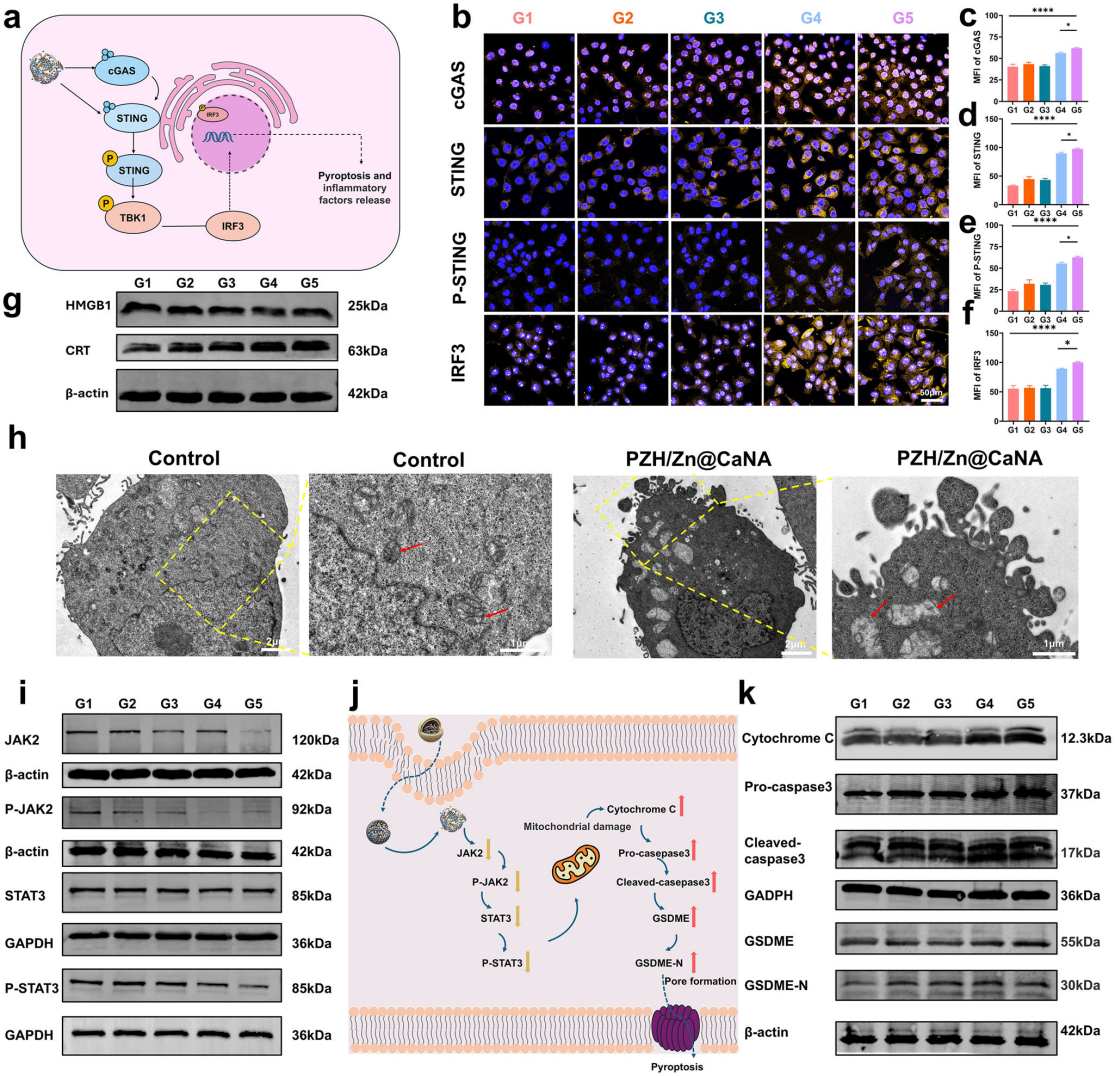
Evaluation of anti‐tumor properties of PZH/Zn@CaNA in vitro. a) Schematic diagram of the mechanism of cGAS‐STING pathway activation by PZH/Zn@CaNA. b) Cellular immunofluorescence staining of cGAS‐STING pathway in Hepa1‐6 cells after different treatments (scar bar: 50 µm). c) Quantitative analysis of fluorescence images of cellular cGAS, d) STING, e) P‐STING, f) IRF3 after different treatments. g) Detection of immunogenic death indicators in treated Hepa1‐6 cells via WB analysis. h) Biological transmission electron microscope (Bio‐TEM) of Hepa1‐6 cells in Control and PZH/Zn@CaNA groups. i) WB analysis of protein expression levels associated with the JAK2‐STAT3 signaling pathway in cellular analysis ontexts. j) Schematic representation of the mechanism by which PZH/Zn@CaNA induces cell death through the JAK2‐STAT3 pathway, in conjunction with the Caspase3‐mediated cellular pyroptosis pathway. k) WB analysis of cellular pyroptosis‐related proteins. (G1: Control; G2: CaNA; G3: PZH@CaNA; G4: Zn@CaNA; G5: PZH/Zn@CaNA). (*p < 0.05, **p < 0.01, ***p < 0.001, ****p < 0.0001).

We have previously identified an immunosuppressive condition in residual tumor tissues from iMWA, and Bimetallic Ca/Zn is a crucial metal ion in metal immunotherapy, particularly relevant for activating the cGAS‐STING immune signaling pathway. To investigate whether PZH/Zn@CaNA could remodel the tumor's immunosuppressive microenvironment, we conducted immunofluorescence staining and quantitative analysis of Hepa1‐6 cells following various treatments. The results indicated that the fluorescence intensity of cGAS, STING, P‐STING, and IRF3 expression was significantly stronger in the PZH/Zn@CaNA group compared to the Zn@CaNA group (**Figure** **4**b–f). This observation can be attributed to both groups containing Bimetallic Ca/Zn, which enhanced intracellular ROS accumulation and increased damaged DNA levels within the cytoplasm. Consequently, this triggered cGAS activation and its downstream STING signaling pathway, leading to cellular pyroptosis along with a substantial release of pro‐inflammatory cytokines that activated systemic immunity (Figure 4a). In comparison to biological transmission electron microscope (Bio‐TEM) images from the control group, treatment with PZH/Zn@CaNA resulted in significant mitochondrial swelling (Figure 4h). Additionally, Zn^2^⁺ and Ca^2^⁺ induced mitochondrial damage while accumulated ROS stimulated the Caspase‐3‐mediated cellular pyroptosis pathway; this led to perforation of hepatocellular carcinoma cells accompanied by the release of pyroptotic vesicles. During this process, ATP associated with immunogenic cell death (Figure S19, Supporting Information) as well as intranuclear HMGB1 were released due to cell membrane fragmentation; notably, CRT protein expression was significantly upregulated to enhance anti‐tumor effects through local activation of ICD (Figure 4g; Figure S20f,g, Supporting Information). This heightened state of ICD subsequently activated the immune system's response. Thus, cellular pyroptosis can synergize with the STING signaling pathway to enhance programmed cell death occurrences intracellularly while simultaneously facilitating extracellular immune reprogramming.

Given the association between the rapid proliferation of iMWA residual tumors and the JAK2‐STAT3 growth signaling pathway, as well as the ability of the encapsulated herbal complex PZH to inhibit tumor growth by suppressing this pathway (Figure 4j; Figure S21, Supporting Information), we aimed to verify whether PZH/Zn@CaNA treatment could effectively inhibit the JAK2‐STAT3 growth signaling pathway and influence the progression of HCC. We extracted proteins from Hepa1‐6 cells subjected to various treatments in vitro, followed by WB assays to quantify both upstream and downstream components related to the JAK2‐STAT3 signaling pathway, including their phosphorylated forms. The results demonstrated a gradual decrease in expression levels of JAK2, P‐JAK2, STAT3, and P‐STAT3. Notably, these reductions were most pronounced in the PZH/Zn@CaNA treatment group, which exhibited the lowest relative content of bands for key target proteins within the JAK2‐STAT3 growth signaling pathway as analyzed through grayscale quantification (Figure 4i; Figure S20h–k, Supporting Information). The inhibition of this signaling pathway significantly suppressed explosive tumor growth associated with iMWA residuals, treatment with PZH/Zn@CaNA resulted in substantial production of ROS and mitochondrial damage. Additionally, WB assays indicated a significant increase in expression levels of target proteins such as CytC, Caspase 3, and GSDME within the Caspase 3‐mediated anaplastic cellular pyroptosis pathway. Quantitative analysis of WB bands yielded statistically significant results (Figure 4k; Figure S20a–e, Supporting Information). These findings suggest that PZH/Zn@CaNA can effectively suppress cell growth and survival while remodeling an immunosuppressive microenvironment in iMWA residual tumors.

### 2.5 In Vivo Validation of Bimetallic Ca/Zn Nanoagonists for Remodeling the Immunosuppressive Microenvironment in iMWA

**Figure 5:**
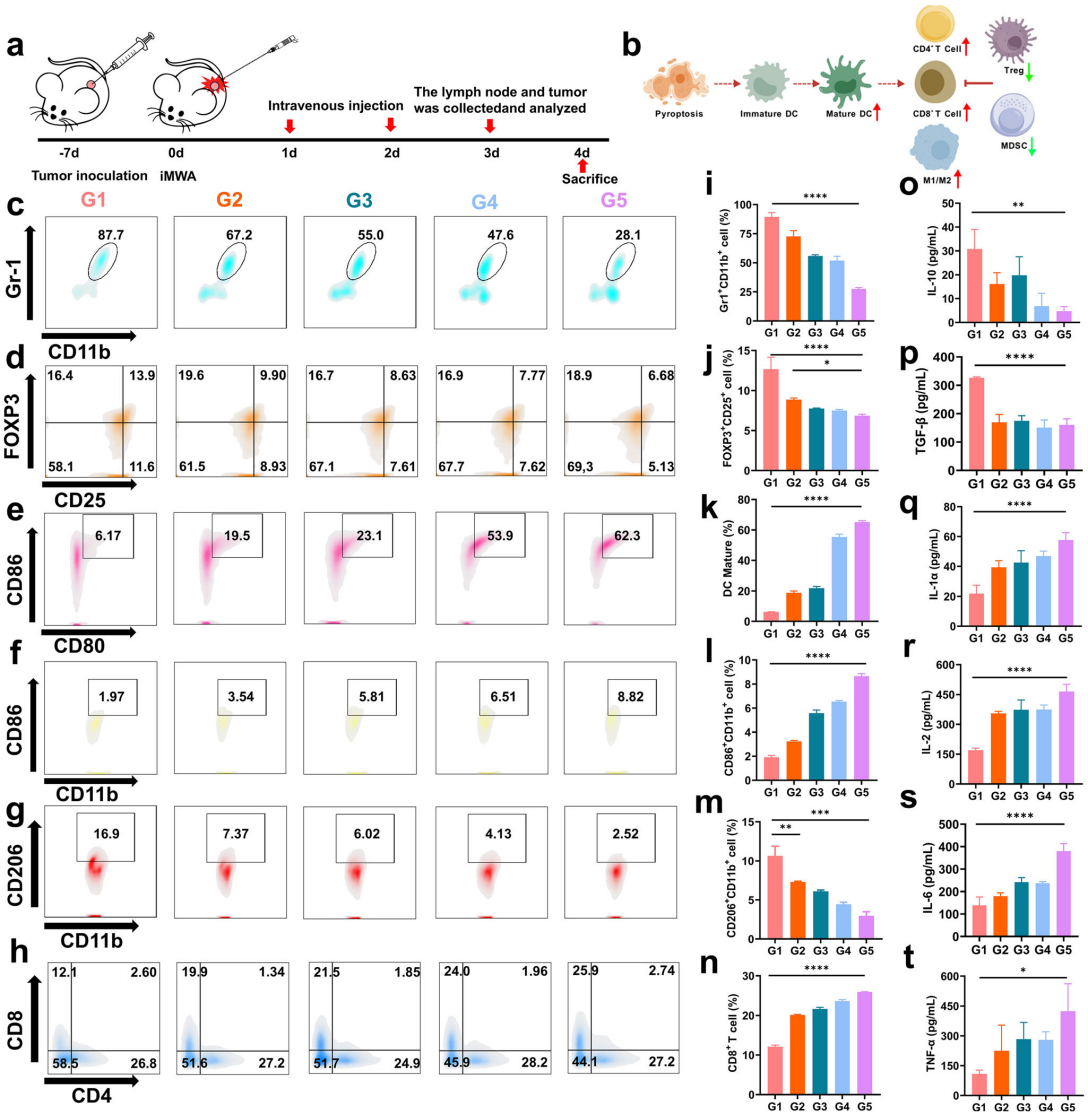
In vivo evaluation of the anti‐tumor properties of PZH/Zn@CaNA. a) Schematic diagram of the experimental design to evaluate the anti‐tumor properties of PZH/Zn@CaNA in vivo and to induce the immune response. b) Schematic diagram of the remodelling of the tumor immune microenvironment by induced cellular pyroptosis. c) Flow cytometry of MDSC, d) Tregs, e) DC, f) M1, g) M2, h) CD8^+^ T cells/CD4^+^ T cells in different treatment groups of mouse tumors, tissues and lymph nodes. i) Quantitative analysis of MDSC, j) Tregs, k) DC, l) M1, m) M2, n) CD8^+^ T cells. o) ELISA quantitative analysis of IL‐10, p) TGF‐β, q) IL‐1α r) IL‐2, s) IL‐6, t) TNF‐α in different treatment groups. (G1: Control; G2: CaNA; G3: PZH@CaNA; G4: Zn@CaNA; G5: /Zn@CaNA). (*p < 0.05, **p < 0.01, ***p < 0.001, ****p < 0.0001).

In order to more precisely validate the regulatory mechanism of PZH/Zn@CaNA on the intratumoral immune system following iMWA, we administered two consecutive treatments of Bimetallic Ca/Zn Nanoagonists to C57BL/6J mice that had undergone iMWA. On the third day post‐treatment, residual tumor tissues and peritumoral lymph nodes were harvested and subjected to FCM for immunophenotypic profiling (**Figure** **5**a). In contrast to the immunosuppressive state observed in residual tumor tissues after pre‐iMWA (Figure 5b), the content of matured CD80^+^CD86^+^ DCs in the lymph nodes of mice treated with PZH/Zn@CaNA was found to be tenfold higher than that in the iMWA group (Figure 5e). Concurrently, the intratumoral immunosuppressive microenvironment induced by iMWA was reversed, specifically, there was a significant reduction in both CD11b^+^Gr‐1^+^ MDSCs and Tregs. Notably, the percentage of MDSCs within residual tumors from mice receiving PZH/Zn@CaNA treatment decreased to 28.1% (Figure 5c,i), while Tregs expression levels diminished to 6.68% (Figure 5d,j). With the decrease in the number of MDSC and Tregs, the secretion levels of the inhibitory cytokines IL‐10 (Figure 5o) and TGF‐β (Figure 5p) were correspondingly reduced, thus attenuating the inhibitory effect on the tumor immune microenvironment. These findings suggest that treatment with PZH/Zn@CaNA effectively mitigates the immunosuppressive microenvironment associated with iMWA and enhances antigen presentation capabilities by promoting DC maturation, thereby stimulating an immune response in vivo. In addition, we conducted FCM quantification to analyze the phenotypes of immune cells, including TAM‐M1, TAM‐M2, and CD3^+^CD8^+^ T cells (Figure 5f–h,l,m). Compared to the control group, different treatments resulted in an increased expression of M1/M2 markers. The degree of intratumoral infiltration by TAM‐M1 cells was significantly enhanced (Figure 5f,l). This finding suggests that TAM‐M1 cells possess a heightened ability to recognize cancer cell signals and deliver “kill signals”. Furthermore, both TAM‐M2 and TAM‐M1 cells exhibit greater sensitivity toward cancer cells and demonstrate improved capabilities in recognizing “kill signals” directed at cytotoxic T cells, thereby triggering a robust immunocidal effect.

The cGAS‐STING signaling pathway has been shown to be inhibited in various cancers. Metalloimmunotherapy utilizing Bimetallic Ca/Zn activates the cGAS‐STING signaling pathway and induces the production of inflammatory cytokines, such as IFN‐γ, IL‐6, and TNF‐α, which inhibit tumor growth and enhance the sensitivity of tumors to checkpoint blockade.^[^ ^28^, ^60^, ^63^ ^]^ Residual tumors following iMWA exhibit a low degree of immune cell infiltration. The fluorescence intensity of proteins associated with the cGAS‐STING signaling pathway was significantly elevated after the application of different treatments (Figure 4b). Notably, the immunofluorescence intensity associated with the cGAS‐STING signaling pathway was found to be highest in Hepa1‐6 cells derived from the PZH/Zn@CaNA group. This observation indicates that PZH/Zn@CaNA group exhibited optimal activation of the cGAS‐STING signaling pathway. The enhanced activity led to an increased release of inflammatory cytokines, including IL‐1α (Figure 5q), IL‐2 (Figure 5r), IL‐6 (Figure 5s), and TNF‐α(Figure 5t), among others (Figure S22, Supporting Information). The released inflammatory cytokines subsequently counteract the immunosuppressive state within residual tumor tissue by activating corresponding immune cells, thereby facilitating the clearance of cancer cells. Furthermore, we found that after PZH/Zn@CaNA treatment, the expression of CRT, a protein related to immunogenicity, was significantly upregulated and exhibited a stronger red fluorescence intensity; while the red fluorescence signal of HMGB1 protein was relatively weak due to its release outside the cells. This result suggests that PZH/Zn@CaNA can promote the death of residual tumor cells after iMWA and effectively activate the adaptive immune response (Figure S23, Supporting Information). This cascading remodeling of the immunosuppressive microenvironment plays a crucial role in controlling uncontrolled growth following iMWA.

### 2.6 In Vivo Validation of the Anti‐Tumor Properties of Bimetallic Ca/Zn Nanoagonists and the Associated Mechanisms Underlying the Inhibition of Hepatocellular Carcinoma Growth

**Figure 6:**
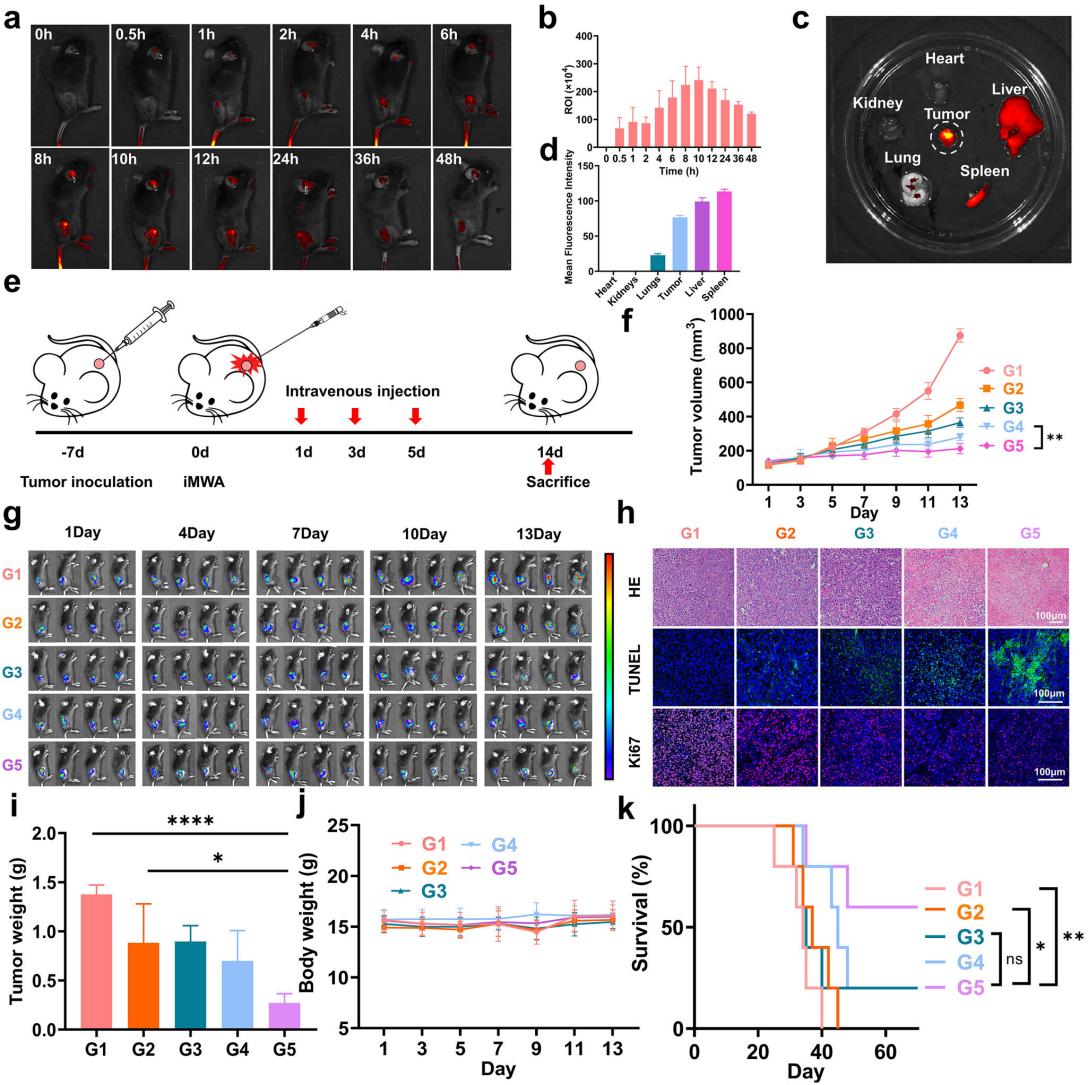
In vivo validation of the anti‐tumor properties of PZH/Zn@CaNA. a) In vivo biofluorescence imaging of DID‐labelled PZH/Zn@CaNA mice. b) Biofluorescence imaging statistics of drug distribution in vivo after fluorescent drug injection in mice. c) Biofluorescence imaging of tumors and major organs 12 h after DID‐PZH/Zn@CaNA injection. d) Biofluorescence imaging analysis of tumors and major organs in mice. e) Schematic diagram of the mouse iMWA model. f) Tumor growth curves of different treatment groups. g) Biofluorescence imaging of mouse tumor volume in different treatment groups (n = 4). h) H&E staining, TUNEL and Ki67 staining of tumor cells in different treatment groups (scar bar: 100 µm). i) Tumor weight of different treatment groups. j) Weight change curves of mice in different treatment groups. k) Survival curves of mice in different treatment groups. (G1: Control; G2: CaNA; G3: PZH@CaNA; G4: Zn@CaNA; G5: PZH/Zn@CaNA). (*p < 0.05, **p < 0.01, ***p < 0.001, ****p < 0.0001).

To verify the excellent biocompatibility of PZH/Zn@CaNA in vivo, blood samples were collected from mice treated with the control group, CaNA, PZH@CaNA, Zn@CaNA and PZH/Zn@CaNA for biochemical and hematological analyses. The results showed no significant toxicity. Additionally, histological examination by H&E staining indicated no damaging lesions in the organs of the mice (Figures S24 and S25, Supporting Information). Further, a short‐term biocompatibility assessment of mice injected only with PZH/Zn@CaNA revealed no significant toxicity compared to healthy mice (Figure S26, Supporting Information). These findings suggest that the synthesized CaNA, PZH@CaNA, Zn@CaNA, and PZH/Zn@CaNA exhibit good biocompatibility. Subsequently, we investigated the metabolism of PZH/Zn@CaNA in vivo through results obtained from fluorescence imaging and fluorescence quantification in mice. The data indicated that, in contrast to the control group, PZH/Zn@CaNA was conspicuously enriched in the iMWA residual tumor 10–12 h post tail vein injection (**Figure** **6**a,b; Figure S27a, Supporting Information). In vivo imaging and quantitative analysis of the major organs (heart, liver, spleen, lung, and kidney) as well as tumor tissues revealed that the enrichment of PZH/Zn@CaNA within the tumor triggered the most intense DID red fluorescence signal (Figure 6c,d; Figure S27c,d, Supporting Information). The intensity of red fluorescence from intratumoral PZH/Zn@CaNA remained robust even after 48 h. This suggests that PZH/Zn@CaNA can be stably delivered to the iMWA residual tumor tissue region where it becomes enriched and releases its therapeutic agent effectively.

To assess the anti‐tumor effect of PZH/Zn@CaNA, mice inoculated with Luc‐Hepa1‐6 cells and treated with iMWA were divided into different groups and treated accordingly (Figure 6e). Tumor volume and body weight changes of mice were recorded every two days, and the bioluminescence signals of tumor tissues after iMWA were monitored by bioluminescence imaging every three days to assess the tumor growth trend. The results of the study showed that the bioluminescent signals of the tumors were attenuated, and their proliferation rate was significantly inhibited under PZH/Zn@CaNA treatment (Figure 6g). At the end of treatment, mice in the PZH/Zn@CaNA‐treated group had the lowest tumor mass (Figure 6i). The body weights of the mice in each group remained stable throughout the experiment, and H&E staining examination of major organs did not show significant toxic responses (Figure 6j; Figure S28, Supporting Information). Additionally, we conducted a survival curve analysis over a 70‐day period and found that mice receiving PZH/Zn@CaNA exhibited the highest survival rate by the conclusion of treatment (Figure 6k). To further substantiate the anti‐tumor effects of PZH/Zn@CaNA, H&E staining performed on tumor tissues at the end of this cycle demonstrated fragmentation of nuclei within tumor cells treated with PZH/Zn@CaNA. These cancer cells displayed loss of their normal structure indicative of programmed cell death. This cellular demise was accompanied by DNA breakage within nuclei and exposure of 3′‐OH termini. To determine the value‐adding properties of cancer cells in anti‐iMWA residual tumors subjected to different treatments, we conducted TUNEL staining and Ki67 staining on tumor tissues. Our findings revealed that following PZH/Zn@CaNA treatment, a significant number of 3′‐OH ends were exposed, which could be stained by fluorescein‐labeled dUTP, resulting in intense green fluorescence observed under a fluorescence microscope. Additionally, Ki67—primarily used as a marker for cells in the proliferative cycle—indicated that the proliferative properties of iMWA residual tumors after PZH/Zn@CaNA treatment were significantly reduced; consequently, weak red fluorescence was detected through fluorescence microscopy (Figure 6h). These results confirm the potent cytotoxic effect of PZH/Zn@CaNA on iMWA residual tumors.

**Figure 7:**
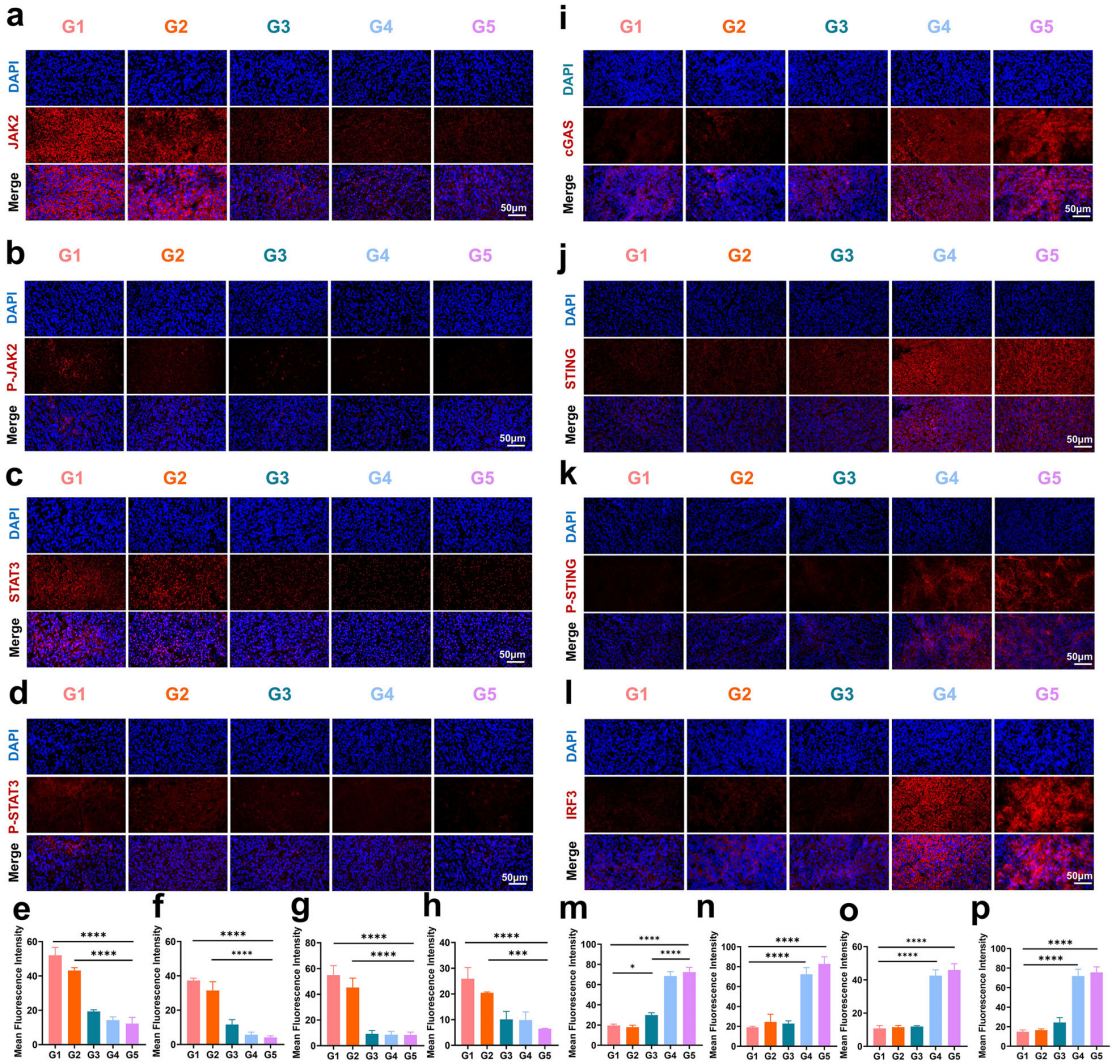
Mechanisms involved in the inhibition of HCC growth in vivo. a) Immunofluorescence images of JAK2, b) P‐JAK2, c) STAT3, d) P‐STAT3 tumors in different treatment groups (scar bar: 50 µm). e) Immunofluorescence image statistics of JAK2, f) P‐JAK2, g) STAT3, h) P‐STAT3 proteins. i) Tumor immunofluorescence of cGAS, j) STING, k) P‐STING, l) IRF3 pathway tumors in different treatment groups (scar bar: 50 µm). m) cGAS, n) STING, o) P‐STING, p) IRF3 immunofluorescence statistics. (G1: Control; G2: CaNA; G3: PZH@CaNA; G4: Zn@CaNA; G5: PZH/Zn@CaNA). (*p < 0.05, **p < 0.01, ***p < 0.001, ****p < 0.0001).

Although the remodeling of the immunosuppressive microenvironment in HCC by PZH/Zn@CaNA represents a crucial strategy for eradicating HCC, the rapid proliferation of residual tumors following iMWA cannot be addressed solely through this remodeling. Instead, it necessitates more robust and effective approaches to inhibit tumor growth.^[^ ^11^, ^13^ ^]^ PZH is recognized for its inhibitory effect on the JAK2‐STAT3 signaling pathway.^[^ ^52^, ^53^, ^54^ ^]^ Previous studies have indicated that the growth of residual tumors following iMWA correlates with the activation of this pathway. Consequently, upon treatment completion, tumor tissues were collected for the assessment of JAK2, P‐JAK2, STAT3, and P‐STAT3 levels. Immunofluorescence and WB analyses demonstrated that PZH@CaNA, Zn@CaNA, and PZH/Zn@CaNA significantly inhibited the expression of JAK2, P‐JAK2, STAT3, and P‐STAT3 in residual tumors post‐iMWA. Notably, the PZH/Zn@CaNA group showed a pronounced decrease in the expression of proteins involved in the JAK2‐STAT3 signaling pathway within tumor tissues (**Figure** **7**a–h; Figure S29, Supporting Information). Moreover, immunohistochemical analysis concerning pyroptosis in tumor tissue revealed elevated expression levels of GSDME within the PZH/Zn@CaNA treatment group—findings consistent with Western blot results related to cellular pyroptosis observed in vitro (Figure S30, Supporting Information). Therefore, PZH/Zn@CaNA enriched in tumor tissues and catabolized to release PZH, Zn^2^⁺ and Ca^2^⁺ can remodel the immunosuppressive microenvironment of tumors, inhibit tumor progression and promote cellular pyroptosis, which is an important reference for managing residual tumors of iMWA.

## 3 Discussion

Microwave ablation therapy is widely used as a preferred local therapeutic strategy for patients with unresectable HCC and early‐stage solitary HCC. However, MWA carries the risk of failing to completely eradicate liver tumors, which may cause cancer cells to shift to explosive growth, leading to spread and metastasis and significantly reducing patient survival. This risk of recurrence is prevalent in surgical resection, radiofrequency ablation, and other localized treatments for HCC. Although several chemo‐immunotherapy strategies have been developed to interrupt the risk of this incomplete treatment, some patients with iMWA still suffer from drug resistance, ineffective treatment, and other adverse effects. Residual cancer cells following iMWA exhibit pronounced heat resistance and demonstrate inhibition of the JAK2‐STAT3 signaling pathway when subjected to stressors within the tumor microenvironment. This response contributes to an enhanced invasive capacity and proliferation rate among these residual cells. In addition, post‐ablation conditions characterized by a weakly acidic microenvironment coupled with elevated oxidative stress levels further promote explosive growth patterns in HCC cells. Notably, the regular yet limited immune activation observed in vivo fails to effectively counteract these highly active states of cancer cells; instead, it may inhibit the immune system's ability to recognize and respond appropriately, resulting in the formation of an immunosuppressive HCC microenvironment. Therefore, employing PZH/Zn@CaNA subsequent to iMWA can effectively inhibit pro‐growth mechanisms while enhancing cellular pyroptosis, while stimulating the intrinsic immunity and remodeling the HCC tumor microenvironment.

We successfully constructed a bimetallic Ca/Zn nanoagonist (PZH/Zn@CaNA), which effectively suppressed tumor activity and remoulded the iMWA‐associated immunosuppressive HCC microenvironment by dual‐mediating inflammatory necrosis and STING pathways. After evaluating the excellent performance of PZH/Zn@CaNA, in vitro experiments revealed that the biomimetic liposome conferred good biocompatibility, which allowed the vector to improve the weakly acidic microenvironment when enriching tumor sites. Simultaneously, the bimetallic ions Ca^2+^ and Zn^2+^ disrupt the balance of oxidative stress in residual cancer cells through calcium deposition in mitochondria, which amplifies the production of ROS in the cytoplasm; and the combination of ROS and PZH (which inhibits the JAK2‐STAT3 signaling pathway) synergistically enhances the inflammation and necrosis of HCC cells. In vivo studies demonstrated that PZH/Zn@CaNA effectively eliminated iMWA subcutaneous tumors, enhanced immunogenic cell death, and triggered a strong anti‐tumor immune response to remodel the immunosuppressive HCC microenvironment. Collectively, this nanoplatform with dual‐mediated cellular pyroptosis and STING pathway can effectively remodel the immunosuppressive HCC microenvironment associated with iMWA and significantly inhibit HCC cell activity, which lays a solid foundation for reducing the risk of recurrence in patients with non‐radical treatment.

## Conflict of Interest

The authors declare no conflict of interest.

## Author Contributions

Y.L., X.L. and K.Y. contributed equally to this work. Y.L. performed conceptualization, data curation, formal analysis, investigation, methodology, visualization, and wrote the original draft. X.L. performed conceptualization, data curation, formal analysis, methodology, wrote, reviewed and edited. K.Y. performed data curation, methodology, investigation, software, and validation. G.Z. performed formal analysis, and methodology. X.Z. performed investigation, and provided software. J.J. performed methodology, and funding acquisition. S.L. and X.W. performed methodology, and formal analysis. Y.Z. and Z.L. performed formal analysis, and visualization. W.M. performed data curation, and formal analysis. D.W. and J.C. performed conceptualization, project administration, supervision, wrote, reviewed & edited, and funding acquisition. All experiments in this study were approved by the ethics committee of Guangxi Medical University Cancer Hospital (approval number: LW2025002), Guangxi Key Laboratory of Early Prevention and Treatment for Regional High Frequency Tumor and Guangxi Medical University Cancer Hospital.

## Supporting information



Supporting Information

## Data Availability

The data that support the findings of this study are available from the corresponding author upon reasonable request.
