# Supplementary material for: Bimetallic Ca/Zn Nanoagonist Remould the Immunosuppressive Hepatocellular Carcinoma Microenvironment Following Incomplete Microwave Ablation via Pyroptosis and the STING Signaling Pathway
Source: Adv Sci (Weinh). 2025 Apr 30;12(23):2500670. doi: 10.1002/advs.202500670 (PMC12199322; doi:10.1002/advs.202500670)
Supplement: Supplementary file 1 — Supporting Information [file ADVS-12-2500670-s001.pdf]

ADVANCED SCIENCE
Open Access

# Supporting Information

for *Adv. Sci.*, DOI 10.1002/advs.202500670

Bimetallic Ca/Zn Nanoagonist Remould the Immunosuppressive Hepatocellular Carcinoma Microenvironment Following Incomplete Microwave Ablation via Pyroptosis and the STING Signaling Pathway

*Yuan Ling, Xiayi Liang, Kangning Yan, Guichun Zeng, Xiaoqi Zhu, Jinghang Jiang, Shaolong Lu, Xiaobo Wang, Yuying Zhou, Zhaoshen Li, Wei Mai, Duo Wang* and Jie Chen**

# Bimetallic Ca/Zn Nanoagonist Remould the Immunosuppressive Hepatocellular Carcinoma Microenvironment Following Incomplete Microwave Ablation via Pyroptosis and the STING Signaling Pathway

*Yuan Ling [a, #], Xiayi Liang [a, #], Kangning Yan [a, #], Guichun Zeng [a], Xiaoqi Zhu [a], Jinghang Jiang [a], Shaolong Lu [a], Xiaobo Wang [a], Yuying Zhou [a], Zhaoshen Li [a], Wei Mai [a], Duo Wang [b,] **, Jie Chen [a,] **

[a] Department of Hepatobiliary Surgery, Department of Medical Ultrasound, Department of Traditional Chinese Medicine, Guangxi Medical University Cancer Hospital, Guangxi Medical University. No. 71 Hedi Road, Nanning 530021, Guangxi, China.

[b] Center of Interventional Radiology & Vascular Surgery, Department of Radiology, Zhongda Hospital, Medical School, Southeast University, Nanjing 210009, China.

* Corresponding author: Department of Hepatobiliary Surgery, Guangxi Medical University Cancer Hospital, Guangxi Medical University. No. 71 Hedi Road, Nanning 530021, Guangxi, China.

** Corresponding author: Center of Interventional Radiology & Vascular Surgery, Department of Radiology, Zhongda Hospital, Medical School, Southeast University, Nanjing 210009, China.

E-mail addresses: jiechen185@163.com (J Chen), wangduo2022@126.com (D Wang).

[#] These authors contributed equally to this work.

**Materials**.

Calcium chloride ($CaCl_2$) and ammonium bicarbonate ($NH_4HCO_3$) were purchased from Sinopharm Chemical Reagent Co., Ltd (Shanghai, China). Zinc Chloride ($ZnCl_2$) (content =99.5%) and 1,2-dioleoyl-sn-glycero-3-phosphate (sodium salt) were purchased from Macklin Biochemical Technology Co., Ltd (Shanghai, China). Anhydrous ethanol and chloroform were purchased from

Kelong Chemical Co., Ltd (Chengdu, China). DSPE-mPEG5000 and 1,2-Dipalmitoyl-sn-glycero-3-phosphocholine (DPPC) were purchased from Ruixi Biotechnology Co., Ltd (Xi'an, China). Cholesterol was purchased from J&K Scientific Co., Ltd (Beijing, China). 1,1'-Dioctadecyl-3,3,3',3'-Tetramethylindodicarbocyanine,4-Chlorobenzenesulfonate Salt (DID) was purchased from UElandy Biotechnology Co., Ltd (Suzhou, China). Pien Tze Huang (PZH) was purchased from Zhangzhou Pien Tze Huang Pharmaceutical Co., Ltd (Fujian, China). Dulbecco's modified Eagle's medium (DMEM), trypsin-EDTA solution, penicillin-streptomycin liquid (penicillin: 10,000.0 U/mL, streptomycin: 10.0 mg/mL) and Hoechst 33342, 2-(4-amidinophenyl)-6-indolecarbamidine dihydrochloride (DAPI) were purchased from Solarbio Science & Technology Co., Ltd (Beijing, China). Triton X-100 and 4% paraformaldehyde were purchased from Biosharp Biotechnology Co., Ltd. Fetal bovine serum (FBS) was obtained from Wisent Biotechnology Co., Ltd (Nanjing, China). CCK8 assay kit was obtained from Beijing Zoman Biotechnology Co., Ltd. Cell-light EdU apollo 567 in vitro kit was obtained from Ribobio Biotechnology Co. Ltd (Guangzhou, China). Mitochondrial membrane potential assay kit with JC-1, adenosine triphosphate (ATP) assay kit, RIPA lysis buffer, phosphatase inhibitor cocktail, western blot (WB) transfer buffer, QuickBlock™ blocking buffer for western blot were obtained from Beyotime Biotechnology Inc. Tricolor Prestained Protein Ladder, PAGE gel rapid preparation kit (10%) and PAGE gel rapid preparation kit (12.5%) were purchased from Epizyme Biomedical Technology Co., Ltd. Goat Anti-Rabbit IgG H&L (Alexa Fluor® 680) was purchased from Abcam plc (UK). ELISA assay kits were obtained from NeoBioscience Technology Co., Ltd (Shenzhen, China).

**Source of antibodies used for WB and immunofluorescence (IF) staining.**

HMGB1 Rabbit monoclonal antibody (A19529), Calreticulin Rabbit monoclonal antibody (A20986), β-actin Rabbit monoclonal antibody (AC038), IRF3 Rabbit polyclonal antibody (A2172), cGAS Rabbit polyclonal antibody (A8335), Cytochrome C Rabbit monoclonal antibody (A4912) and Active + pro caspase-3 Rabbit monoclonal antibody (A19654) were purchased from ABclonal Biotechnology Co., Ltd (Wuhan, China). STAT3 Rabbit polyclonal antibody (381552) and Phospho-STAT3 (Tyr705) Rabbit polyclonal antibody (251611) were purchased from Zen-Biotechnology Co., Ltd (Chengdu, China). Phospho-JAK2 (Tyr931) antibody (AF3024), JAK2 antibody (AF6022) and Phospho-TMEM173/STING (Ser366) antibody (AF7416) were purchased from Affinity

Biosciences Ltd (America), DFNA5/GSDME polyclonal antibody (Cat. No. 13075-1-AP) and TMEM173/STING Polyclonal antibody (Cat. No. 19851-1-AP) were purchased from Proteintech Group, Inc (America).

**Cell lines and animals.**

Luc-Hepa1-6 cells and Hepa1-6 cells (mouse liver cancer cells) were purchased from Zhejiang Meisen Technology Co., LTD. Luc-Hepa1-6 cells and Hepa1-6 cells were cultured in DMEM containing 10% fetal bovine serum and 1% penicillin-streptomycin at 37°C and 5% $CO_2$ in the cell incubator. C57BL/6J mice (4 weeks old) were obtained from the Animal Experiment Center of Guangxi Medical University. All animal experiments were conducted in accordance with the ethical guidelines formulated by the Ministry of Health and approved by the Animal Experiment Center of Guangxi Medical University.

**Ethical approval.**

The experimental animals were provided by the Laboratory Animal Center of Guangxi Medical University. The experimental procedures were carried out in strict accordance with the standards set by the Animal Protection Supervision Committee. Ethical approval was granted by the ethics committee of Guangxi Medical University Cancer Hospital (approval number: LW2025002), and the endpoint of the experimental study was when the tumor volume reached 1500 $mm^3$.

**Establishment of sublethal heat stress cell model and iMWA mouse model.**

To simulate incomplete microwave ablation (iMWA) therapy, we established an in vitro non-lethal heat therapy model [1-4]. Hepa1-6 cells were seeded in a 6-well plate at a density of $2.0 \times 10^5$ cells/well in 1.0 mL DMEM and cultured overnight. The medium was replaced with FBS-DMEM preheated to 40°C before heat treatment. The plate was subsequently immersed in a water bath maintained at 40°C for a duration of 15 min. After heating, the plates were returned to the incubator for cultivation. All cell experiments were performed with these pre-treated cells. In a mouse iMWA model, a subcutaneous tumor implant was successfully established on the dorsal side of the mouse by inoculating $8\times10^6$ cells. Once the tumor reached a critical size, with a maximum diameter of approximately 0.8 cm, microwave ablation was performed using a No. 19 single electrode (from Lide Electronics Co., Ltd, Mianxang, China). The electrode has an active tip of 1 cm and is inserted

percutaneously until its tip reaches the midpoint of the long axis of the tumor. The ablation process was performed for 30 seconds at a power setting of 7W to achieve partial necrosis of the target tumor in a controlled manner [3, 5, 6].

**Synthesis of PZH/Zn@CaNAs.**

PZH/Zn@CaNAs were synthesized by employing the classical one-pot gaseous diffusion method [7-9]. Firstly, a 250 mL conical flask containing 100.0 mL of anhydrous ethanol, 150.0 mg of $CaCl_2$, 20 mg of PZH, and 20 mg of $ZnCl_2$ were added into a closed vessel. In addition, 5g of $NH_4HCO_3$ was placed in the same sealed container, and the closed container was placed in a constant temperature water bath at 42 °C for 72 h. During this time, $NH_4HCO_3$ was decomposed by heat to produce $CO_2$ gas, which flowed into the mixture containing $CaCl_2$ to produce calcium carbonate nanoparticles encapsulating PZH and $Zn^{2+}$. The mixture was collected, centrifuged repeatedly at 12,000 rpm, and the precipitate was extracted; the nanoparticles were washed twice with anhydrous ethanol and dried at 42°C for 2 h. The precipitate, DSPE-mPEG5000, DPPC, and cholesterol were dispersed in chloroform in the ratio of 20mg: 20mg: 10mg: 1mg: 5ml. After magnetic stirring for 6h, the chloroform was removed by vacuum rotary evaporation to obtain DSPE-mPEG5000 and cholesterol-modified nanoparticles (PZH/Zn@CaNAs); they were resuspended with PBS and placed in refrigerator at 4°C for storage.

**Characterizations.**

Transmission electron microscopy (TEM) and element mapping were observed by a JEOL JEM-1200EX transmission electron microscope with an acceleration voltage of 100 kV (Tokyo, Japan) and a FEI Tal os F200X double transmission field gun with an acceleration voltage of 200 kV (FEI, USA), respectively. Fourier transform infrared spectroscopy (FTIR) was recorded on Nicolet6700 FT-IR spectrometer (Thermo Nicolet, USA). X-ray photoelectron spectroscopy (XPS) uses Thermo escalab 250XI (Thermo Scientific, USA). Zeta potential and dynamic light scattering (DLS) measurements were performed on the Zetasizer Nano S (Malvern, 6UK). High-performance liquid chromatography-tandem mass spectrometry (LC-MS/MS) was performed with Agilent 1290II-6460 (California, USA). Inductively coupled plasma (ICP) is obtained by ThermolCPOES7200 (Thermo Fisher, USA).

**Release of PZH from PZH/Zn@CaNA.**

To investigate the degree of release of PZH from the drug, 2 mL of PZH/Zn@CaNA (6 mg/mL) was placed in two dialysis bags (MWCO = 3500 Da). The dialysis bags were then soaked in 60.0 mL PBS with pH 5.4 and 7.4, respectively, and incubated at 37°C. At a predetermined time, 3 mL of the supernatant of each sample was collected, and the corresponding PBS was replenished to the original volume. PZH release was detected by liquid chromatography-mass spectrometry.

**Release of $Ca^{2+}$ and $Zn^{2+}$ from PZH/Zn@CaNA.**

To investigate the degree of ion release of PZH/Zn@CaNA, first, 12 mg of PZH/Zn@CaNA was dissolved in PBS at pH 7.4 and placed into dialysis bags (MWCO = 3500 Da), and both ends were closed and sealed. Then, the dialysis bags were immersed in 60.0 mL PBS with pH 5.4 and 7.4, respectively, and incubated at 37°C. At a predetermined time, 3 mL of the supernatant from each sample was collected and then 3 mL of the corresponding PBS was added to the original volume. Inductively coupled plasma optical emission spectrometry (ICP-OES) was used to detect the release of $Ca^{2+}$ and $Zn^{2+}$.

**Detection of the cellular internalization ability of PZH/Zn@CaNA.**

To evaluate the uptake of PZH/Zn@CaNA by cells. PZH/Zn@CaNA was stirred with DID overnight to obtain DID-PZH/Zn@CaNA. Hepa1-6 cells were inoculated into 24-well plates containing cell crawls at a density of $5\times10^4$ cells per well and incubated overnight. Then, the old medium was discarded and 1.0 mL of DID-PZH/Zn@CaNA (50ug/mL) was added for co-incubation. At various time points (0 h, 0.5 h, 1 h, 2 h, 4 h, 6 h, and 8 h), the crawlers were subjected to two washes with PBS before being fixed for a duration of 15 minutes. Subsequently, they were stained with DAPI for 20 minutes. After sealing and drying the crawlers, cellular visualization was performed using laser confocal scanning microscopy (CLSM) with an excitation wavelength (λex) of 644 nm and an emission wavelength (λem) of 663 nm. Additionally, cells were cultured and treated with drugs as previously described. Following digestion with trypsin, the cells were collected for flow cytometry analysis (FCM). This analysis quantified changes in fluorescence intensity of DID within the cells. The resulting data were processed and analyzed using Flowjo v10 software.

**In vitro cytotoxicity.**

To evaluate the effect of PZH/Zn@CaNA on Hepa1-6 cell viability in vitro, Hepa1-6 cells were first inoculated into 96-well plates at a concentration of $5 \times 10^3$ cells per well and cultured overnight. Subsequently, the cells were treated with 100 μL of FBS-DMEM medium containing various concentrations of PZH/Zn@CaNA for a duration of 24 h. Following this incubation period, 100 μL of a working solution consisting of 10% CCK8 (DMEM: CCK8 = 90:10) was added to each well. The absorbance was measured at a wavelength of 450 nm using an enzyme-labeled instrument. The cytotoxicity of PZH/Zn@CaNA towards normal hepatocytes (THLE-2) was assessed using the same methodology. In addition, the EdU method was employed to further investigate cellular proliferation. Hepa1-6 cells were seeded onto cell slides within 24-well plates at a density of $5 \times 10^4$ cells per well and cultured overnight. The medium was then replaced with varying concentrations of PZH/Zn@CaNA, followed by the addition of 200 μL medium containing 50 μM EdU to each well for incubation over two hours. After washing twice with PBS, each well received an application of fixative solution (incubated at room temperature for thirty minutes with paraformaldehyde at a concentration of 4%, followed by five minutes with glycine at a concentration of 2 mg/mL). Subsequently, each well was treated with penetrant (0.5% Triton X-100) and incubated for ten minutes before being washed again with PBS. Thereafter, each well received an addition of 200 μL Apolo staining reagent diluted to a concentration suitable for use and incubated in darkness at room temperature for thirty minutes. Samples were then stained using Hoechst33342 for fifteen minutes and sealed with an anti-fluorescence quencher prior to imaging via CLSM (Confocal Laser Scanning Microscopy). Cells labeled with azide dye conjugate number567 are classified as EdU-positive cells.

**EdU assay for monitoring cell proliferation.**

Cell proliferation was analyzed following the instructions provided by the Cell-Light EdU Apollo 567 external kit. Hepa1-6 cells were inoculated in a 24-well plate at a density of $5 \times 10^4$ cells per well and cultured overnight. The cells were categorized into five groups: G1 (control), G2 (CaNA), G3 (PZH@CaNA), G4 (Zn@CaNA), and G5 (PZH/Zn@CaNA). Subsequently, 200 μL of medium containing 50 μM EdU was added to each well and incubated for two hours. After washing twice with PBS, 200 μL of cell fixative (4% paraformaldehyde) was added for incubation at room temperature for 30 minutes, followed by the addition of 200 μL of glycine solution (2 mg/mL) to each well for an additional five minutes. Next, 200 μL of penetrant solution (0.5% Triton X-100)

was introduced and incubated for ten minutes. Following another wash with PBS, each well received 200 μL of the Apolo dyeing reaction solution diluted to a concentration of 1× and was incubated at room temperature in the dark for thirty minutes. The samples were then stained with Hoechst 33342 for fifteen minutes and sealed using an anti-fluorescence quencher. Finally, images of the samples were captured using CLSM. Cells labeled with azide dye conjugate number 567 are defined as EdU-positive cells.

**Analysis of intracellular $Ca^{2+}$.**

To investigate the intracellular degradation and release of PZH/Zn@CaNA, Hepa1-6 cells were seeded at a density of $5\times10^4$ cells per well in 24-well plates containing cell crawlers at the bottom and cultured overnight. Depending on the experimental group, various drugs were administered to treat the cells accordingly. Following washing with PBS, the cells were stained for intracellular $Ca^{2+}$ using the Fluo-4 AM probe, incubated for 1 h, and subsequently blocked with an anti-fluorescence quencher. Imaging was conducted using CLSM.

**Detection of intracellular pH.**

To assess intracellular pH changes, Hepa1-6 cells were inoculated into 24-well plates containing cell crawlers at a density of $5\times10^4$ cells/well and incubated overnight. After treatment with different drugs, the cells were incubated with the pH probe BCECF AM (Beyotime Biotechnology Inc. S1006) stain for 1 h and protected from light according to the procedure described in the instruction manual, and the resulting cell crawls were observed by CLSM.

**Mitochondrial membrane potential assay.**

To determine the changes in mitochondrial membrane potential, Hepa1-6 cells were inoculated at a density of $5\times10^4$ cells/well into 24-well plates containing cell crawls. After the cells were attached to the wall, different drug treatments were administered. The JC-1 fluorescent probe was added to the cells at a dilution of 1:200, incubated for 20 minutes at 37°C in the absence of light, washed three times with PBS and then blocked with an anti-fluorescence quencher, and the subsequent samples were imaged by CLSM. At high mitochondrial membrane potential, JC-1 aggregated in the mitochondrial matrix to form polymers (aggregates) and produced red fluorescence recorded at the excitation wavelength of 525 nm and the emission wavelength of 590

nm. However, when the mitochondrial membrane potential was low, JC-1 could not aggregate in the mitochondrial matrix, and then JC-1 was a monomer that could produce green fluorescence with an excitation wavelength of 490 nm and an emission wavelength of 530 nm.

**Detecting reactive oxygen species (ROS).**

To assess the production of ROS in the cells, which were stained with the ROS stain DCFH-DA probe. Hepa1-6 cells were inoculated at a density of $5\times10^4$ cells/well into 24-well plates containing cell culture media and cultured overnight. Different drug treatments were added according to the different groups. The cells were washed twice with PBS and stained with ROS stain DCFH-DA probe working solution (DCFH-DA: DMEM=1:500). The cells were then incubated in a cell culture incubator (37°C, 5% $CO_2$), protected from light for 30 min, washed twice with PBS and then stained with Hoechst 33342 for 15 min. The resulting cell crawls were imaged by CLSM and statistically analysed accordingly.

**Immunofluorescence analysis.**

To test the effect of PZH/Zn@CaNA on the internal growth pathway of cancer cells, Hepa1-6 cells were inoculated into 24-well plates at a density of $5\times10^4$ cells/well and cultured overnight. Different drugs were added and treated according to the different groups. The cells were washed twice with PBS, fixed with 4% paraformaldehyde for 15 minutes, washed three times with PBS, incubated with 0.5% (v/v) Triton X-100 for 20 minutes at room temperature, washed again with PBS, and then sealed with rapid containment solution for 30 minutes at room temperature. The containment solution was discarded and the cells were washed three times with PBS and then added to primary antibodies at 4°C for overnight incubation. The corresponding primary antibodies were: cGAS (1:200), STING (1:200), P-STING (1:200) and IRF3 (1:200). The samples were then washed three times with PBS and incubated with fluorescence coupled secondary antibody working solution at room temperature for 1 h. After washing, the samples were stained with Hoechst 33342 and protected from light for 15 minutes, and the resulting samples were imaged and analyzed by CLSM.

**Western Blot.**

To evaluate the changes in protein expression in tumor cells after PZH/Zn@CaNA treatment, Hepa1-6 cells were inoculated into 6-well plates at a density of $2.0 \times 10^5$ cells/well and cultured

overnight. Intracellular proteins were extracted after administration of different drug treatments, extraction with RIPA lysis buffer containing protease and phosphatase inhibitors. Protein quantification was performed using a microvolume UV-visible spectrophotometer (Thermo Scientific™NanoDrop™One, USA). A medium amount of protein (40-60 μg) was taken from each group, electrophoresed on a 10%, 12.5% PAGE gel separation (200 V, 36 min) and transferred to a PVDF membrane (400 mA, 88 min). The PVDF membrane was blocked with QuickBlock™ blocking buffer for 30 minutes and the PVDF membrane was incubated with primary antibodies at 4°C overnight. The primary antibodies used were HMGB1 (1:1000), CRT (1:1000), β-actin (1:2000), Cytochrome C (1:1000), Active + pro-caspase-3 (1: 2000), GSDME (1:2000), GAPDH (1:2000), P-JAK2 (1:1000), JAK2 (1:1000), P-STAT3 (1:1000) and STAT3 (1:1000). After washing three times with TBST, the PVDF membrane was incubated with Alexa Fluor® 680-conjugated fluorescent secondary antibody working solution at room temperature and protected from light for 1 hour. After washing again with TBST, the membrane was detected using an Odyssey DLx near-infrared dual-colour laser imaging system (Gene Company Limited, China).

**Assay for intracellular ATP levels.**

To detect changes in intracellular ATP levels, Hepa1-6 cells were inoculated into 6-well plates at a density of $2.0 \times 10^5$ cells/well and cultured overnight. According to the different groups, different drugs were added for treatment. Following two washes with PBS, 200 μL of lysis solution was added to the sample. The sample was then centrifuged at 12,000 rpm for 5 minutes, after which the supernatant was collected for further experimentation. Then, 100 μL of ATP assay working solution was added to the test wells using a special coated-well plate (BS-MP- 96B, Biosharp, China), and the plate was left at room temperature for 3-5 minutes to deplete all the background ATP. Then 20 μL samples were added to the test wells and immediately detected using a luminometer (Thermo, USA).

**Animal experiment.**

C57BL/6J mice (4 weeks old) were obtained from the Animal Experiment Center of Guangxi Medical University. Briefly, $8.0 \times 10^6$ Luc-Hepa1-6 cells were injected into the right dorsum of C57BL/6J mice, and the experiments were terminated when the subcutaneous tumors reached a

volume of approximately 1500 $mm^3$. Tumor volume was calculated using the formula: =length × $width^2$ /2

**Metabolism of PZH/Zn@CaNA in vivo.**

To investigate the metabolism of the drug in the organism. DID dye and DID-PZH/Zn@CaNA at 50ug/mL was injected into 4-week-old C57BL/6J tumor-bearing mice (n=3) via tail vein. In vivo biofluorescence imaging was performed at 0 h, 0.5 h, 1 h, 2 h, 4 h, 6 h, 8 h, 10 h, 12 h, 24 h, 36 h, and 48 h, respectively. In addition, tumors, hearts, livers, spleens, lungs and kidneys of C57BL/6J mice were collected for biofluorescence imaging at 12 h after injection of DID dye and DID-PZH/Zn@CaNA.

**Investigation of the immunological effects of PZH/Zn@CaNA in vivo.**

The mice were subjected to iMWA (parameters: 7W 30s) until tumor formation, and the Luc-Hepa1-6 mice were divided into 5 groups (n=6): G1: control; G2: CaNA; G3: PZH@CaNA; G4: Zn@CaNA; G5: PZH/Zn@CaNA. The drug was then administered intravenously via the tail for 2 consecutive days (40 mg/kg). On day 3, lymph nodes, tumors and serum were collected from the mice for response testing. The serum was subjected to ELISA for cytokine expression. The expression of cytokines (IL-1α, IL-2, IL-6, IL-10, IL-12p70, TNF-α, IL-10, TGF-β, IFN-γ) in the serum of mice was measured according to the instructions of the ELISA kit. Tumors and lymph nodes were collected for flow cytometry to determine the immunophenotypes of DC cells, T cells, Treg cells, myeloid-derived suppressor cells (MDSC) and macrophages. The colour schemes for the corresponding immunophenotypes are as follows:

DC cells: CD11c-APC-H, CD86-FITC-A, CD80-PE-A

T cells: CD3 PerCP-Cy5.5-A, CD4-FITC-H, CD8-PE-H

Treg cells: CD45 APC-Cy7-A, CD3PerCP-Cy5.5-A, CD4 BV510-A, FOXP3 APC-A

Myeloid-derived suppressor cells (MDSC): CD45-FITC-H, CD11b-PE-H, Gr-1-APC-H

Macrophages: M1 CD11b-PE-A, CD86-FITC-A; M2: CD11b-PE-A, CD206-APC-A

**In vivo anti-tumor evaluation in mice.**

To evaluate the anti-tumor effect of PZH/Zn@CaNA activation in vivo, Luc-Hepa1-6 tumor-bearing mice were randomly divided into 5 groups (n=4) after incomplete microwave ablation: G1: control; G2: CaNA; G3: PZH@CaNA; G4: Zn@CaNA; G5: PZH/Zn@CaNA. Tail vein administration was performed every other day (40 mg/kg). Starting on day 1, tumor size was measured every 2 days with a caliper (formula: length × width$^2$ /2) and the corresponding tumor volume was calculated. Biofluorescence imaging was performed every 3 days to visualise tumor growth in real time. At the end of the 14-day observation cycle, the mice were sacrificed, and the tumor tissues were collected and weighed. The tumor tissues were embedded and analysed by H&E staining, terminal deoxynucleotidyl transferase-mediated dUTP nick-end labelling (TUNEL) staining and Ki-67 staining. Meanwhile, to verify the effects of PZH/Zn@CaNA treatment on the JAK2-STAT3 pathway in mouse tumors and the immune effects produced in vivo, tumor tissues from different treatment groups were collected for fluorescent staining of sections, including P-JAK2 (1:200), JAK2 (1:200), P-STAT3 (1:200), STAT3 (1:200), cGAS (1:200), STING (1:200), P-STING (1:200) and IRF3 (1:200) were observed by fluorescence microscopy and the corresponding images were taken. To further verify the safety of the treatment, we collected major organs such as heart, liver, spleen, lung and kidney for H&E observation. In addition, 30 tumor-bearing mice were reconstructed and after undergoing incomplete microwave ablation treatment, they received similar drug treatments (n=5) and were subjected to survival analysis and observed for 70 days. Tumor volume of 1500 mm$^3$ or premature death of the mice was used as the endpoint of the experimental design.

**Biofluorescence imaging of tumors in mice.**

Biofluorescence imaging (PerkinElmer, USA) was used to monitor the volume and size of Luc-Hepa1-6 tumors in mice. Tumor-bearing C57BL/6J mice were injected intraperitoneally with 20 μL of DTZ-Luc1 substrate (Meisen CTCC), anaesthetised with methyl bromide (0.1 mL/10 g body weight) and photographed in the loading chamber 3-5 min later. Photographs of the mice were analysed using bioluminescence living imaging (Living imaging version 4.4, Caliper Life Sciences) and ImageJ software.

**Hemolysis test.**

Appropriate amounts of mouse blood cells were collected and incubated with different concentrations of PZH/Zn@CaNA (0, 50, 100, 150, 200, 250 and 300 μg/mL). Meanwhile, PBS and purified water were used as negative and positive controls, respectively. The supernatant was centrifuged at 3500 rpm for 10 minutes and the absorbance at 540 nm was measured using an enzyme marker. The hemolysis rate was calculated as: hemolysis rate (%) = (A-A0) / (A∞-A0) × 100%. Here, A, $A_0$ and $A_\infty$ are the absorbance of the groups of experimental medicine, PBS and DI water, respectively.

**Biological safety evaluation.**

To confirm the biosafety of the nanomedicine, the healthy C57BL/6J mice were randomly divided into 5 groups (n=3): PBS, CaNA, PZH@CaNA, Zn@CaNA, and PZH/Zn@CaNA. The drug was administered via tail vein on days 1, 2, and 3, and then the mice were killed on day 14. Whole blood and serum were collected from different groups. In addition, the healthy C57 BL/6J mice were injected with PZH/Zn@CaNA into the tail vein and divided into three groups (n=3). Whole blood and serum were collected 1, 3 and 7 days after injection. The above samples were sent for routine blood, liver and kidney function tests. The corresponding indices were white blood cell count (WBC), red blood cell count (RBC), platelet count (PLT), hemoglobin count (HGB), lymphocyte count (Lymph), absolute neutrophils, alanine aminotransferase (ALT), aspartate aminotransferase (AST), total bilirubin (TBIL), UREA, creatinine (CREA) and uric acid (UA). At the same time, major organs (heart, liver, spleen, lungs and kidneys) were collected for H&E.

**Statistical analysis.**

Statistical analyses were performed using ImageJ software and GraphPad Prism software (version 8.01), and statistical comparisons between groups were performed using one- and two-way analysis of variance (ANOVA) or t-test. Values are expressed as mean ± standard deviation (SD). $P < 0.05$ was considered statistically significant, *$P < 0.05$, **$P < 0.01$, ***$P < 0.001$, ****$P < 0.0001$ was considered highly significant.

**Supplementary figures**

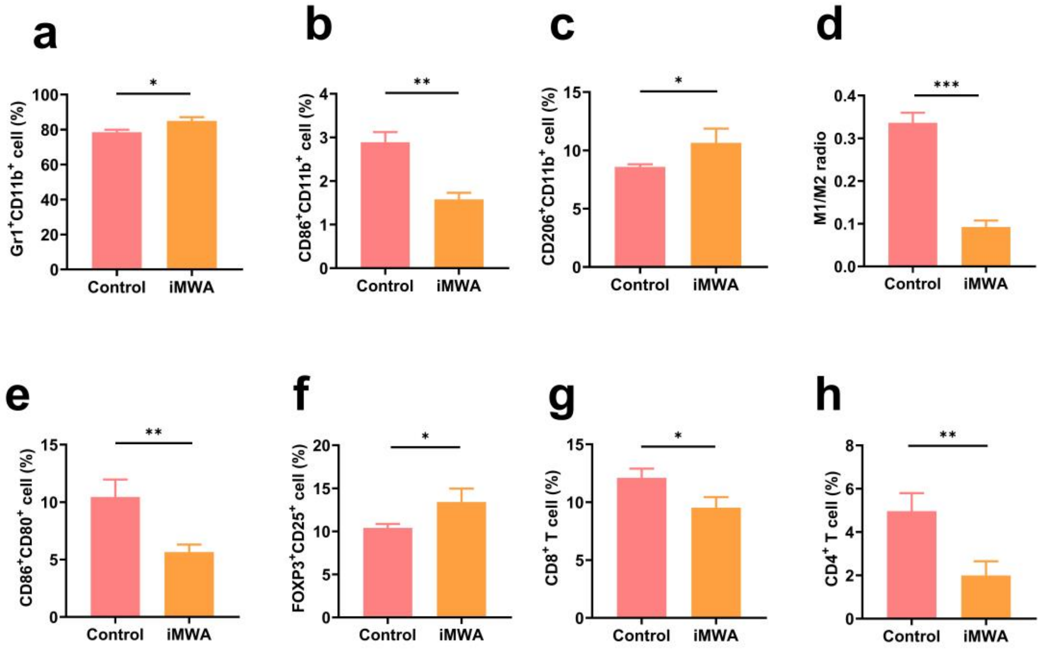


**Figure S1.** (a) Quantitative analysis of MDSC, (b) M1 macrophages, (c) M2 macrophages, (d) the ratio of M1 to M2, (e) dendritic cells (DC), (f) regulatory T cells (Treg), (g) $CD8^{+}$ T cells, and (h) $CD4^{+}$ T cells were conducted using flow cytometry across different treatment groups. (*p < 0.05, **p < 0.01, ***p < 0.001, ****p < 0.0001).

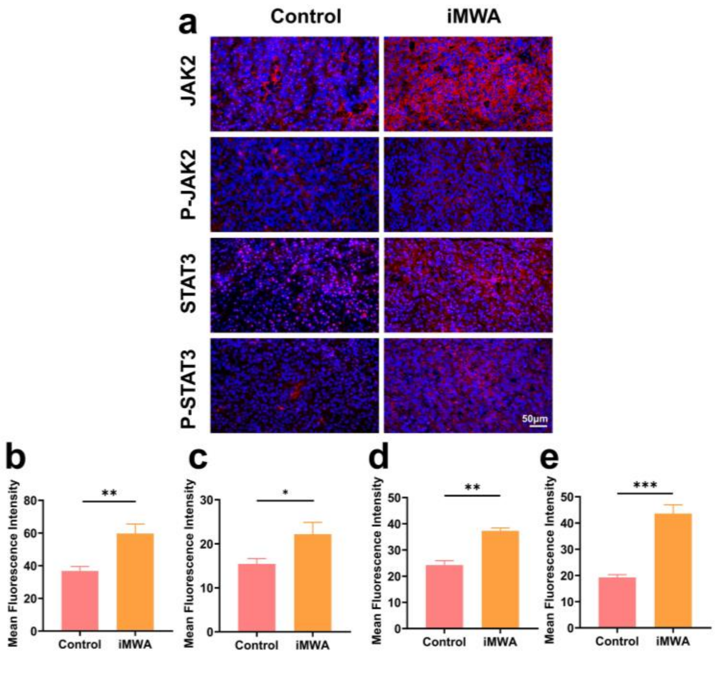


**Figure S2.** (a) Immunofluorescence images of JAK-2, P-JAK2, STAT3, P-STAT3 proteins after

different treatments and quantitative analysis of fluorescence intensity of (b) JAK-2, (c) P-JAK2, (d) STAT3, (e) P-STAT3 fluorescence images (scar bar: 50 μm). (*p < 0.05, **p < 0.01, ***p < 0.001, ****p < 0.0001).

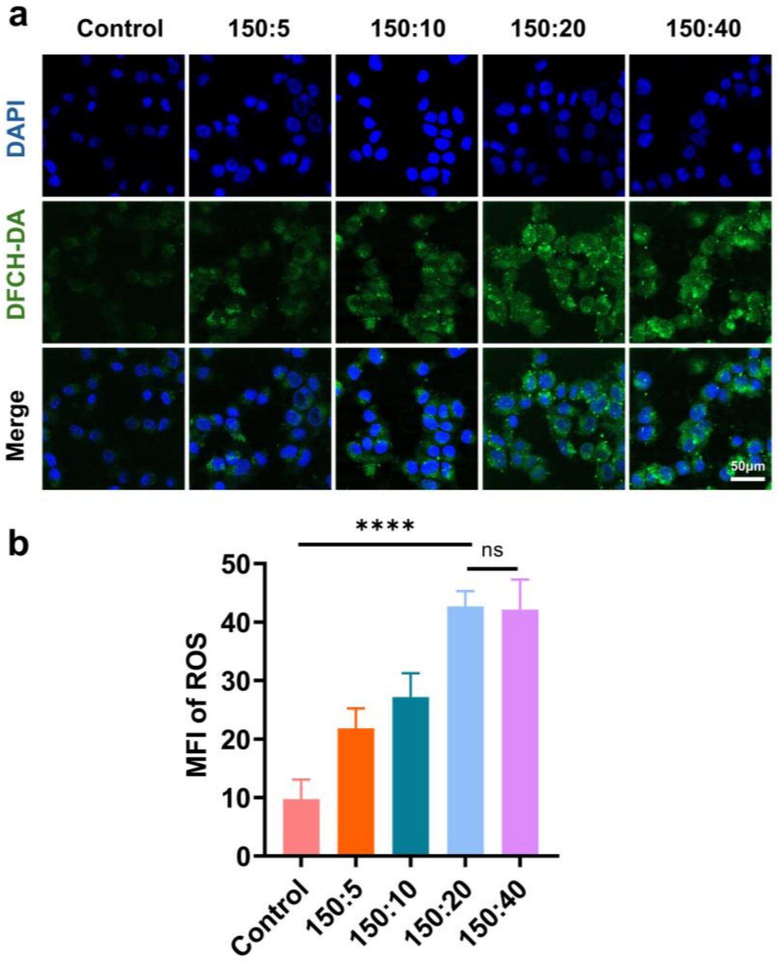


**Figure S3.** (a) CLSM images of ROS production in Hepa1-6 cells after different proportion of bimetallic ions (scar bar: 50 μm). (b) Quantitative statistics of ROS in Hepa1-6 cells from different proportion of bimetallic ions. (*p < 0.05, **p < 0.01, ***p < 0.001, ****p < 0.0001).

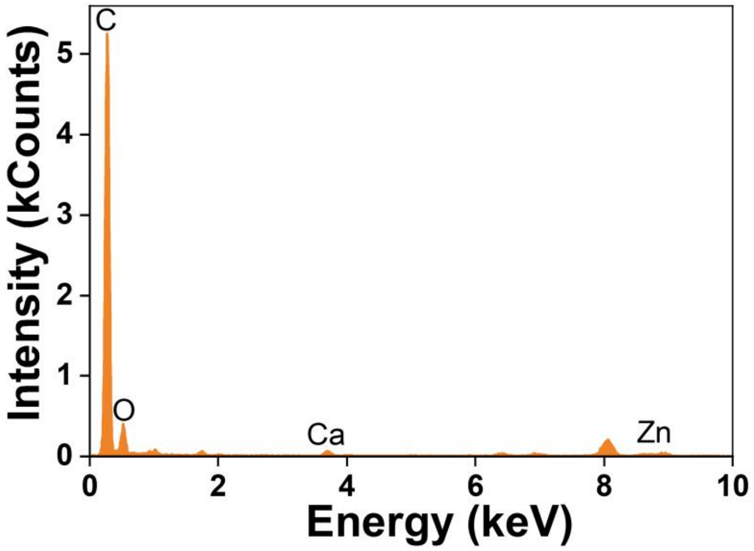


**Figure S4.** EDS elemental analysis of PZH/Zn@CaNA.

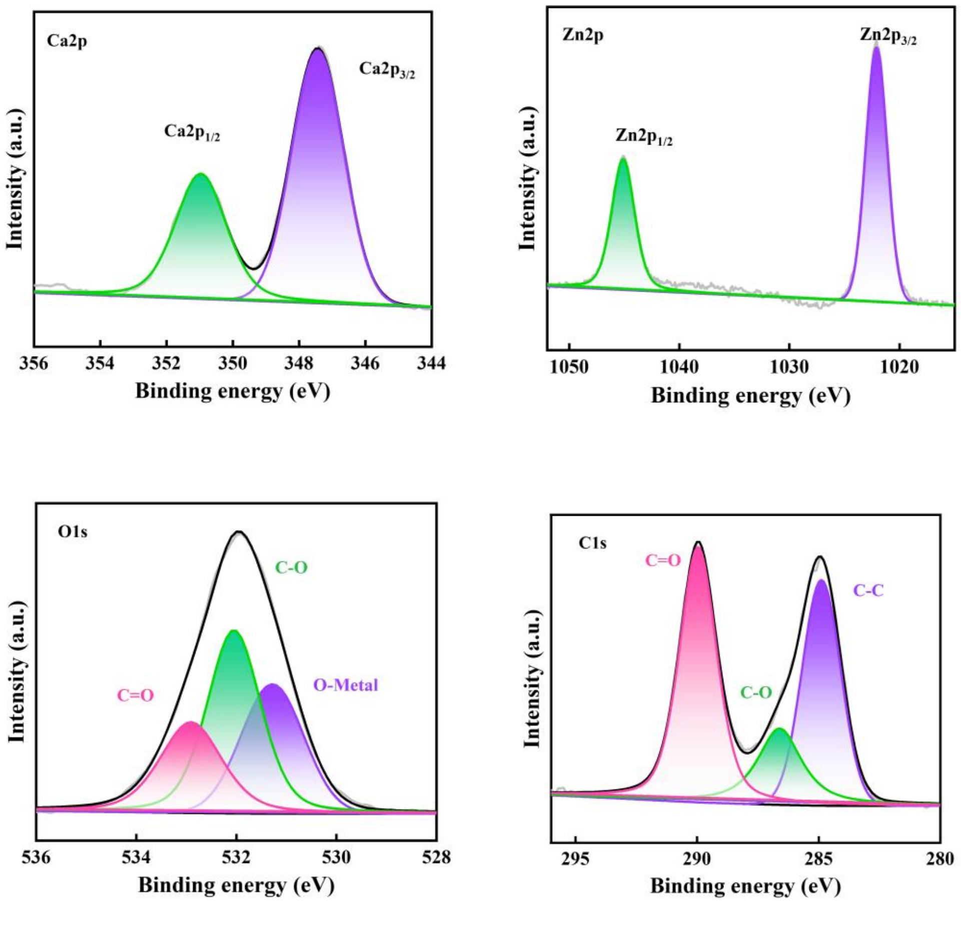


**Figure S5.** XPS spectra of (a) Ca2p, (b) Zn2p, (c) O1s, (d) C1s of PZH/Zn@CaNA.

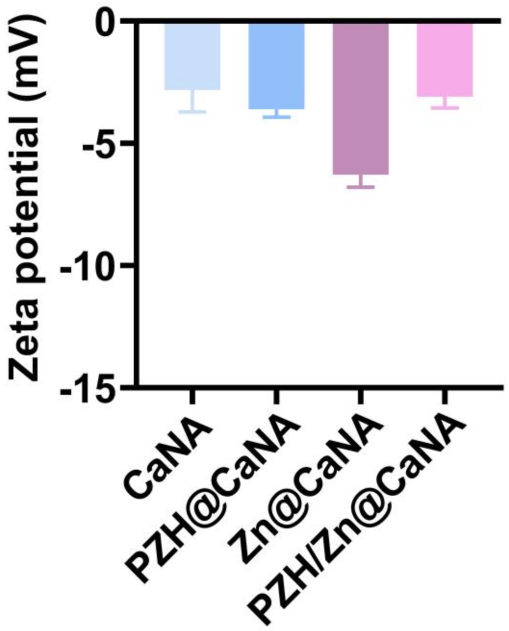


**Figure S6.** Zeta potentials of CaNA, PZH@CaNA, Zn@CaNA and PZH/Zn@CaNA.

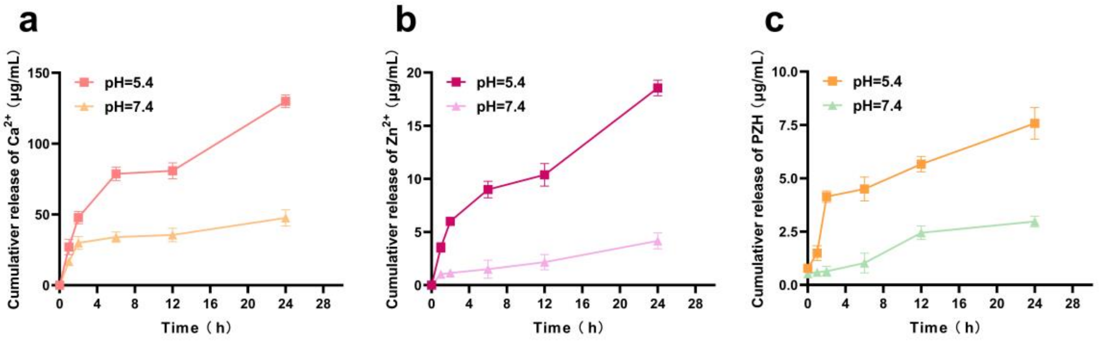


**Figure S7**. (a) Drug release concentration profiles on $Ca^{2+}$, (b) $Zn^{2+}$ and (c) PZH in pH=5.4 and 7.4.

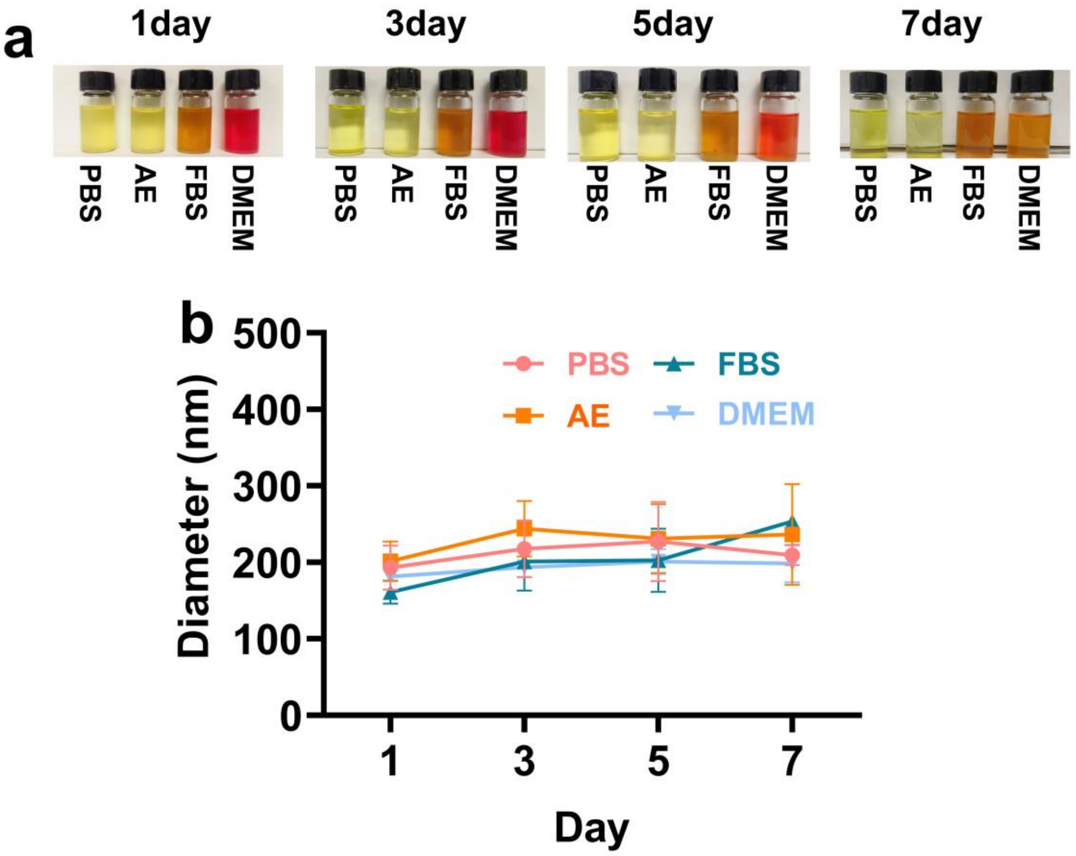


**Figure S8.** Stability analysis. (a) Digital images of PZH/Zn@CaNA in solution and (b) particle size variation curves with time in different media. (PBS: Phosphate Buffer Solution, FBS: Foetal Bovine Serum, AE: Anhydrous Ethanol, DMEM: Dulbecco's Modified Eagle Medium).

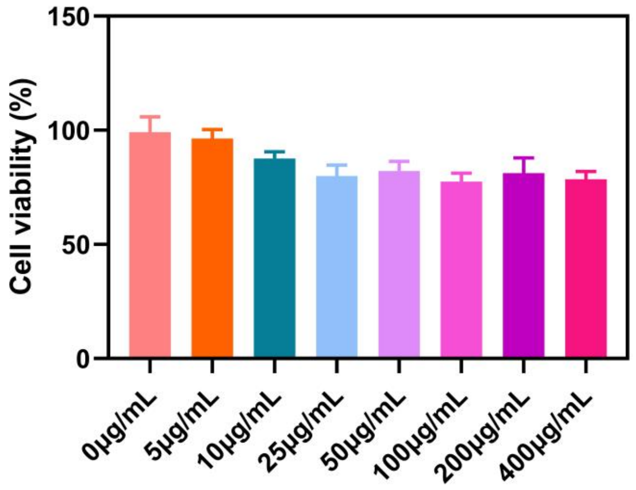


**Figure S9.** Cellular viability of THLE-2 in normal hepatocytes by different concentrations of PZH/Zn@CaNA.

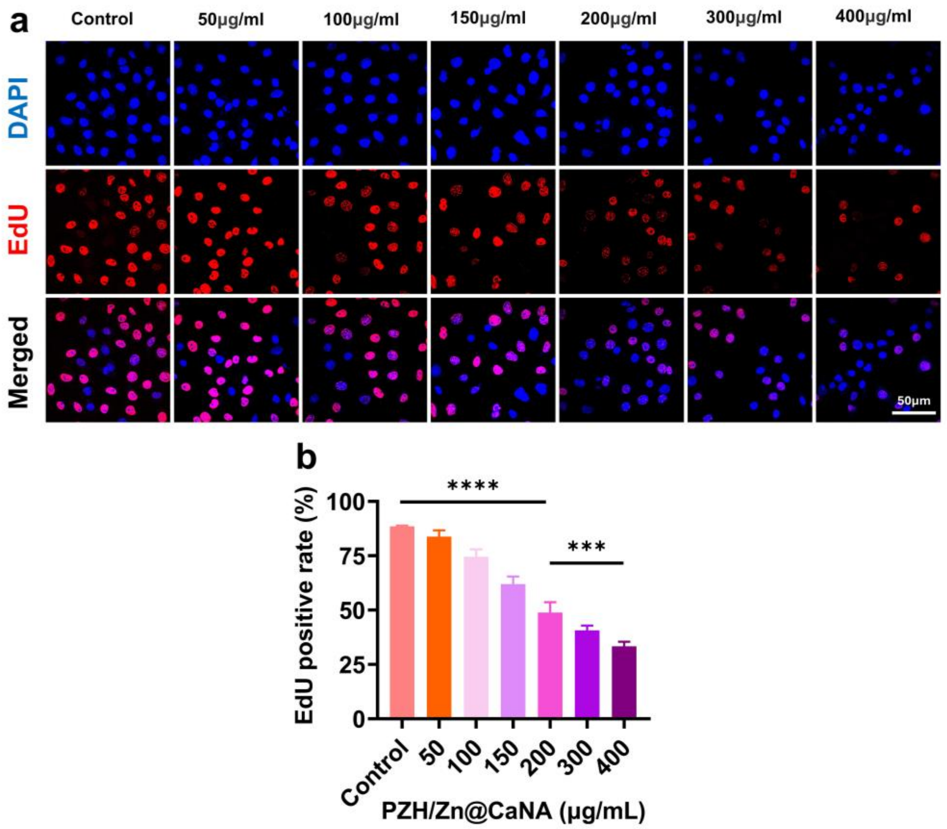


**Figure S10.** (a) EdU cytofluorescence and (b) fluorescence quantification of different concentrations of PZH/Zn@CaNA on Hepa1-6 cells (scar bar: 50 μm). (*$p < 0.05$, **$p < 0.01$, ***$p < 0.001$, ****$p < 0.0001$).

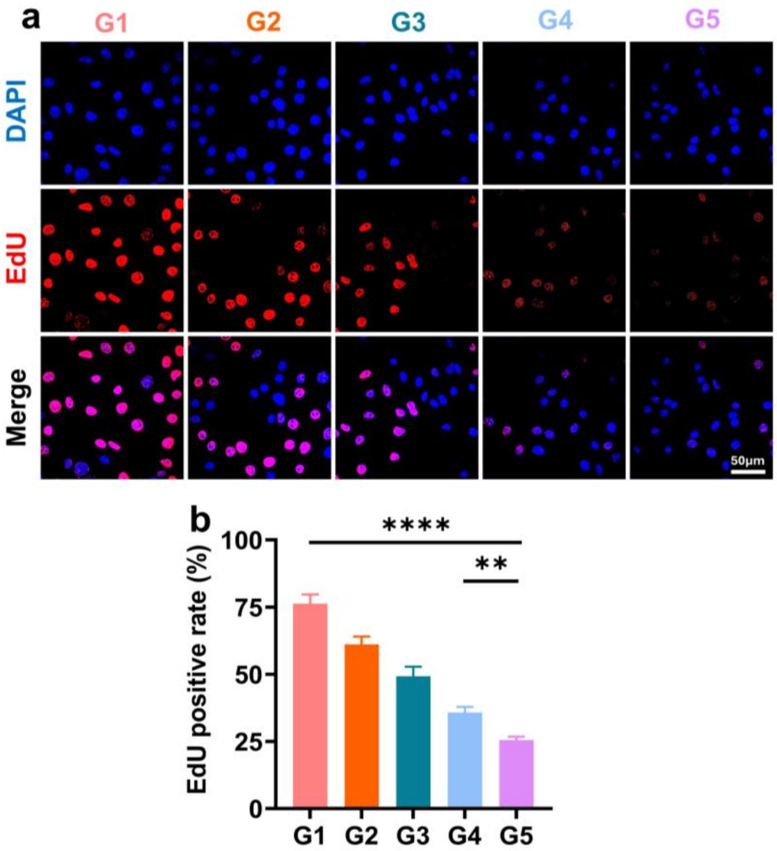


**Figure S11.** (a) EdU-stained CLSM images of proliferative capacity of Hepa1-6 cells in different treatment groups and (b) EdU positive cell ratio (scar bar: 50 μm). (G1: Control; G2: CaNA; G3: PZH@CaNA; G4: Zn@CaNA; G5: PZH/Zn@CaNA). (*p < 0.05, **p < 0.01, ***p < 0.001, ****p < 0.0001).

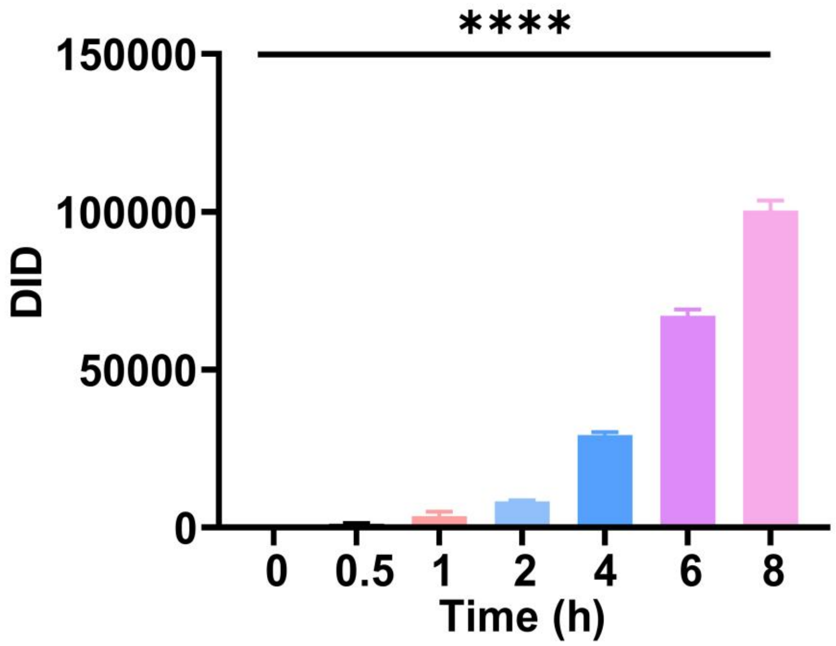


**Figure S12.** Flow cytometry statistics of cells swallowing DiD-PZH/Zn@CaNA at different times. (*$p < 0.05$, **$p < 0.01$, ***$p < 0.001$, ****$p < 0.0001$).

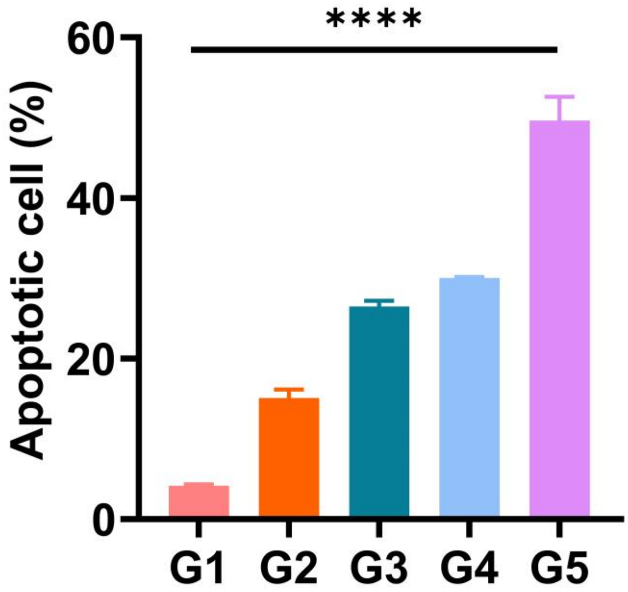


**Figure S13.** Flow cytometry statistics of apoptotic cells in different treatment groups. (G1: Control; G2: CaNA; G3: PZH@CaNA; G4: Zn@CaNA; G5: PZH/Zn@CaNA). (*$p < 0.05$, **$p < 0.01$, ***$p < 0.001$, ****$p < 0.0001$).

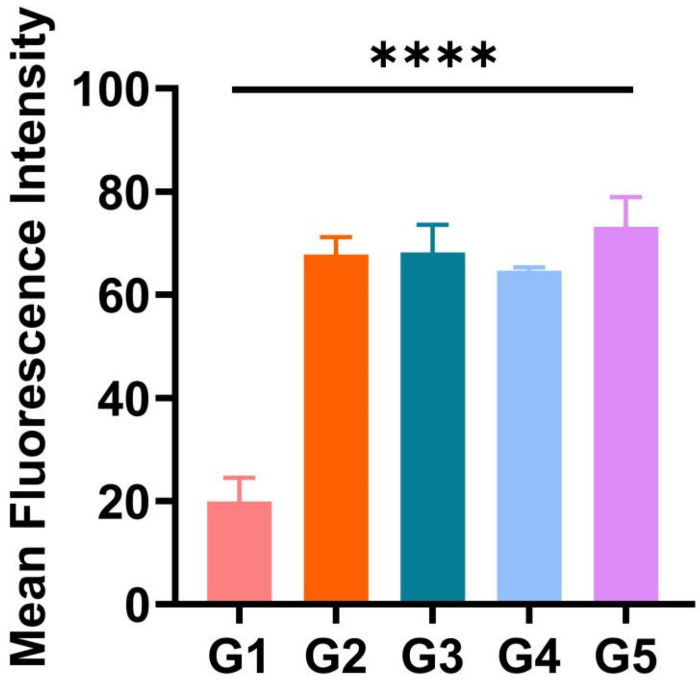


**Figure S14.** Quantitative statistics of intracellular pH fluorescence in Hepa1-6 cells from different treatment groups. (G1: Control; G2: CaNA; G3: PZH@CaNA; G4: Zn@CaNA; G5: PZH/Zn@CaNA). (*p < 0.05, **p < 0.01, ***p < 0.001, ****p < 0.0001).

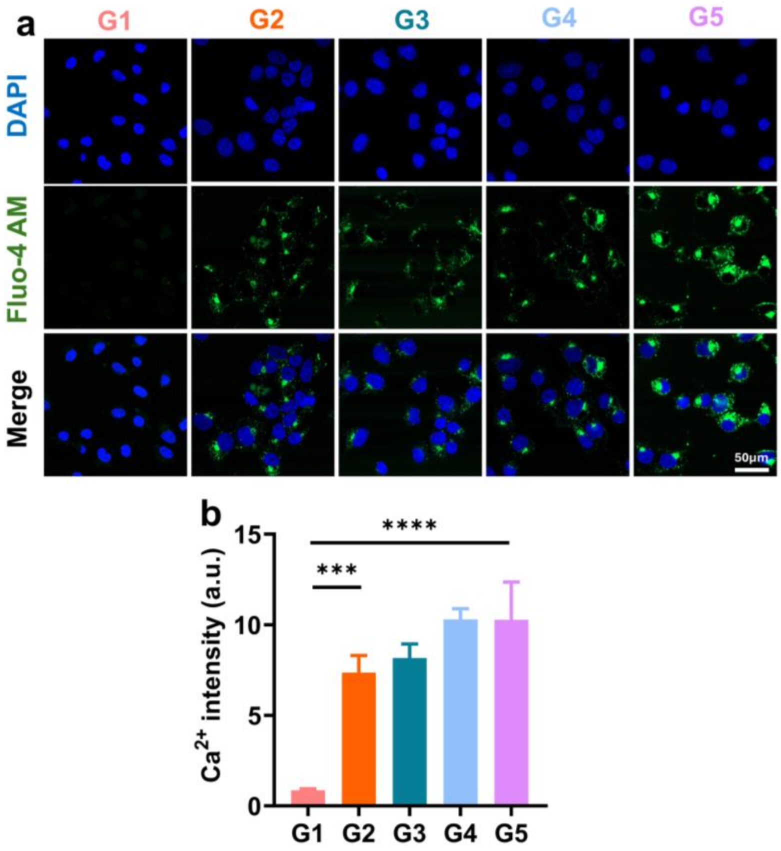


**Figure S15.** (a) CLSM of the intracellular $Ca^{2+}$ probe Fluo-4 AM staining and (b) fluorescence statistics of fluorescence images in different treatment groups (scar bar: 50 μm). (G1: Control; G2: CaNA; G3: PZH@CaNA; G4: Zn@CaNA; G5: PZH/Zn@CaNA). (*p < 0.05, **p < 0.01, ***p < 0.001, ****p < 0.0001).

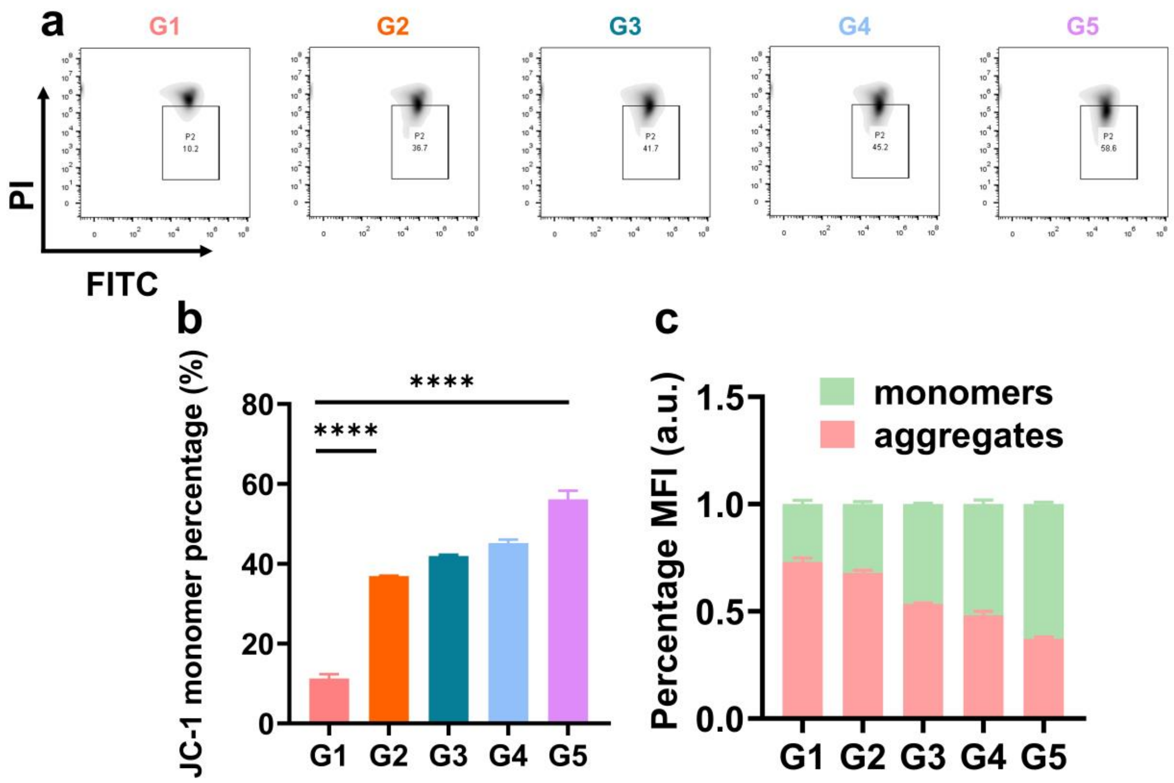


**Figure. S16.** (a) Flow cytometry was employed to analyze and (b) quantify the mitochondrial membrane potential through JC-1 staining fluorescence intensity in Hepa1-6 cells derived from various treatment groups. (G1: Control; G2: CaNA; G3: PZH@CaNA; G4: Zn@CaNA; G5: PZH/Zn@CaNA). (c) Quantitative analysis of mitochondrial JC-1 fluorescence staining images. (*p $< 0.05$, **p $< 0.01$, ***p $< 0.001$, ****p $< 0.0001$).

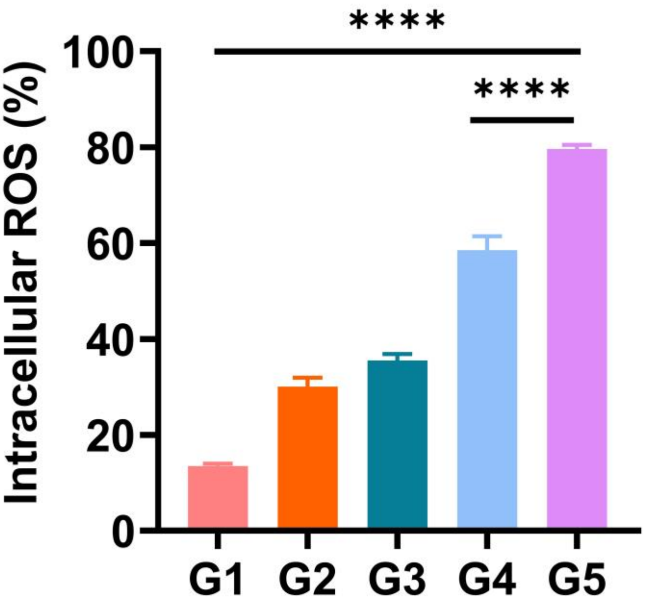


**Figure S17.** Flow cytometry analysis of ROS generation in different treatment groups. (G1: Control; G2: CaNA; G3: PZH@CaNA; G4: Zn@CaNA; G5: PZH/Zn@CaNA). (*p < 0.05, **p < 0.01, ***p < 0.001, ****p < 0.0001).

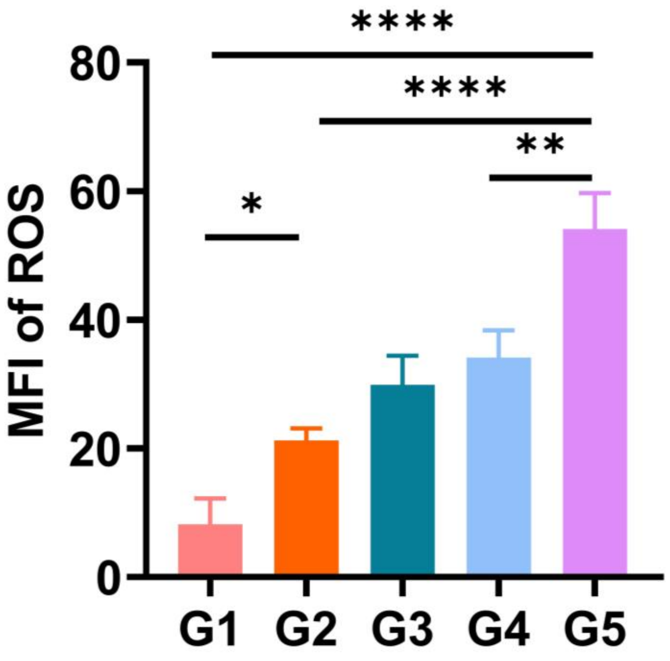


**Figure S18.** (a) Quantitative statistics of ROS in Hepa1-6 cells from different treatment groups. (G1: Control; G2: CaNA; G3: PZH@CaNA; G4: Zn@CaNA; G5: PZH/Zn@CaNA). (*p < 0.05,

**p < 0.01, ***p < 0.001, ****p < 0.0001).

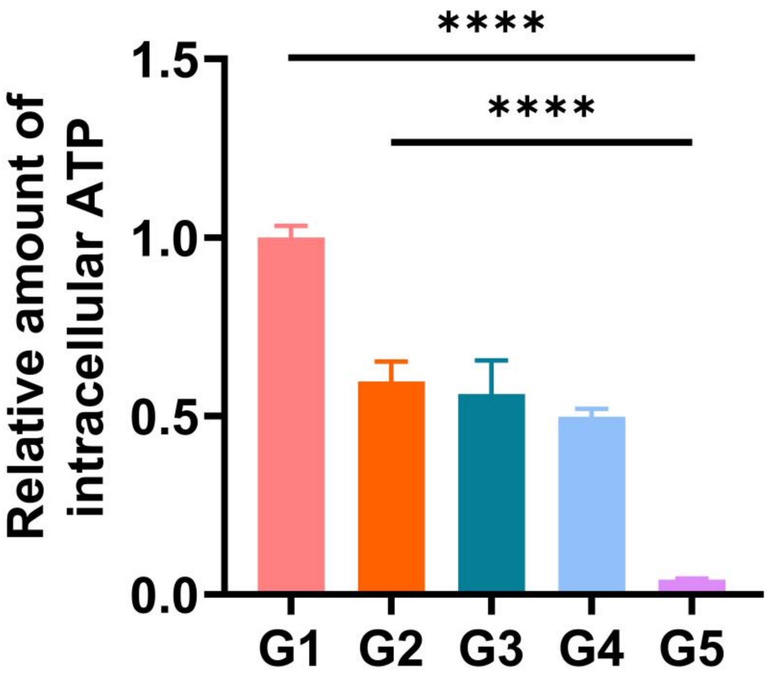


**Figure S19.** Intracellular ATP statistics of different treatment groups. (G1: Control; G2: CaNA; G3: PZH@CaNA; G4: Zn@CaNA; G5: PZH/Zn@CaNA). (*p < 0.05, **p < 0.01, ***p < 0.001, ****p < 0.0001).

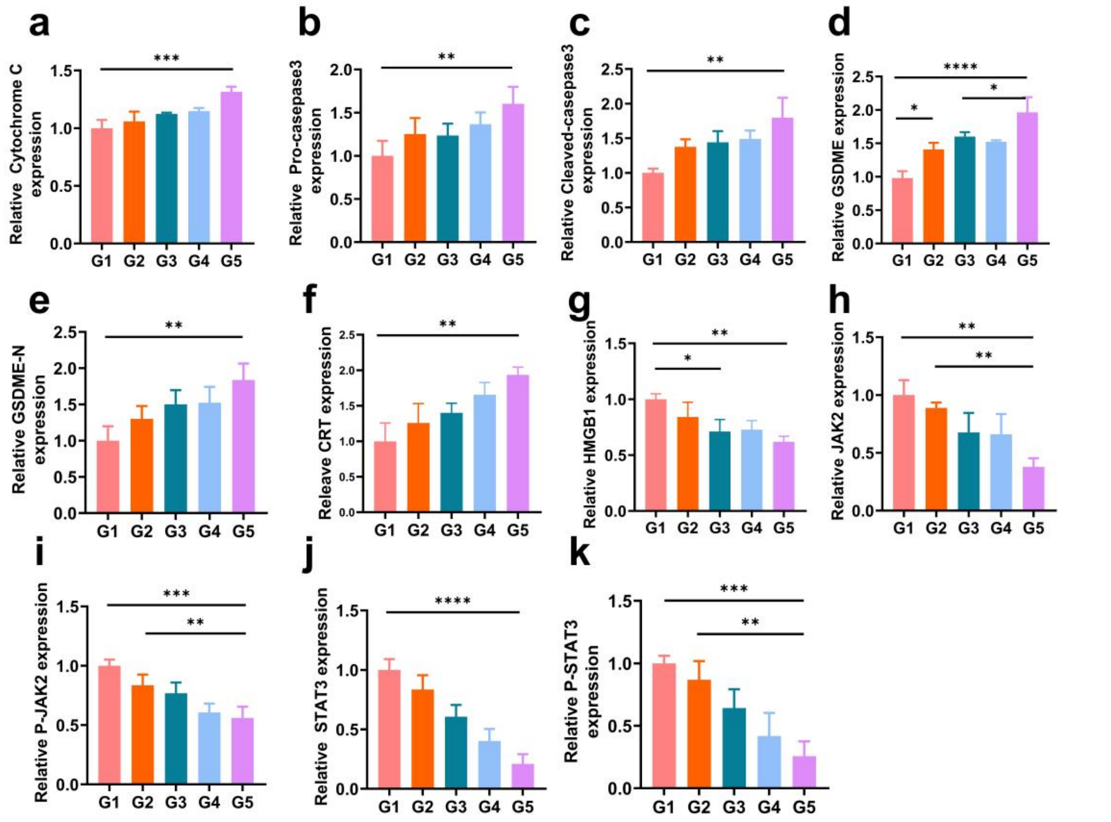


**Figure S20.** Quantitative analysis of the grayscale intensity of protein bands. (a) Relative expression levels of the Hepa1-6 cell proteins Cytochrome C, (b) Pro-casepase3, (c) Cleaved-casepase3, (d) GSDME, (e) GSDME-N, (f) CRT, (g) HMGB1, (h) JAK2, (i) P-JAK2, (j) STAT3 and (k) P-STAT3 after different treatments. (*$p < 0.05$, **$p < 0.01$, ***$p < 0.001$, ****$p < 0.0001$).

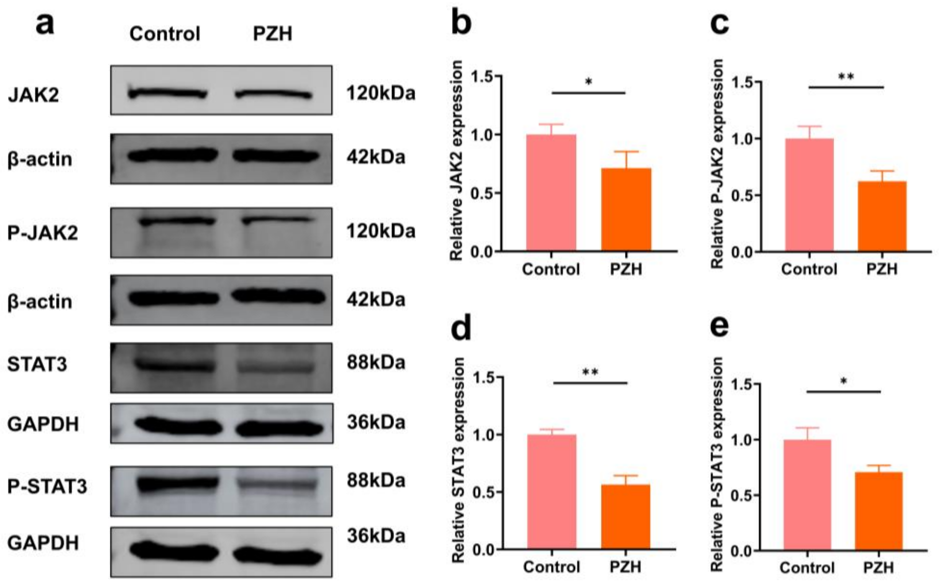


**Figure S21.** (a)WB analysis of protein expression levels associated with the JAK2-STAT3 signaling pathway in cellular analysis and quantitative analysis of WB images of (b) JAK2, (c) P-JAK2, (d) STAT3, (e) P-STAT3. (*$p < 0.05$, **$p < 0.01$, ***$p < 0.001$, ****$p < 0.0001$).

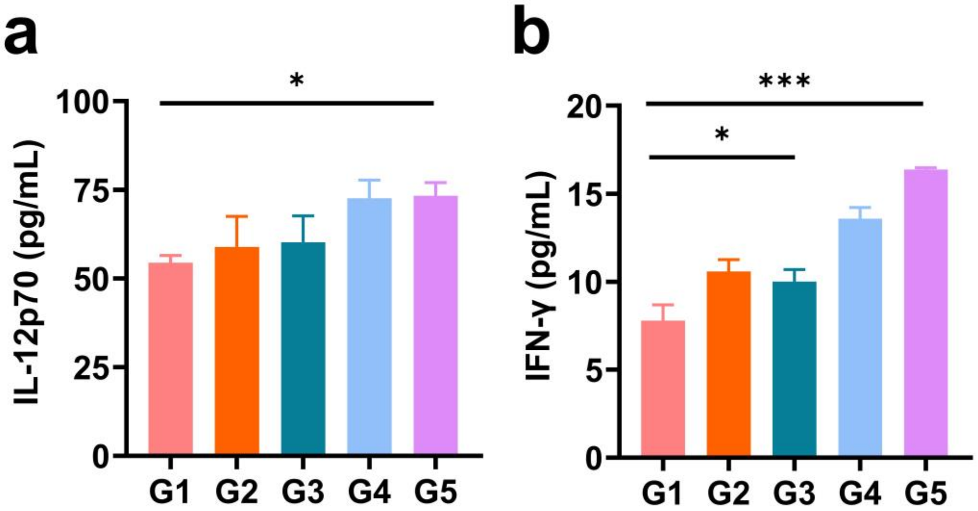


**Figure S22.** ELISA statistics of (a) IL-12p70, (b) IFN-γ in different treatment groups of C57BL/6J mice. (G1: Control; G2: CaNA; G3: PZH@CaNA; G4: Zn@CaNA; G5: PZH/Zn@CaNA). (*$p < 0.05$, **$p < 0.01$, ***$p < 0.001$, ****$p < 0.0001$).

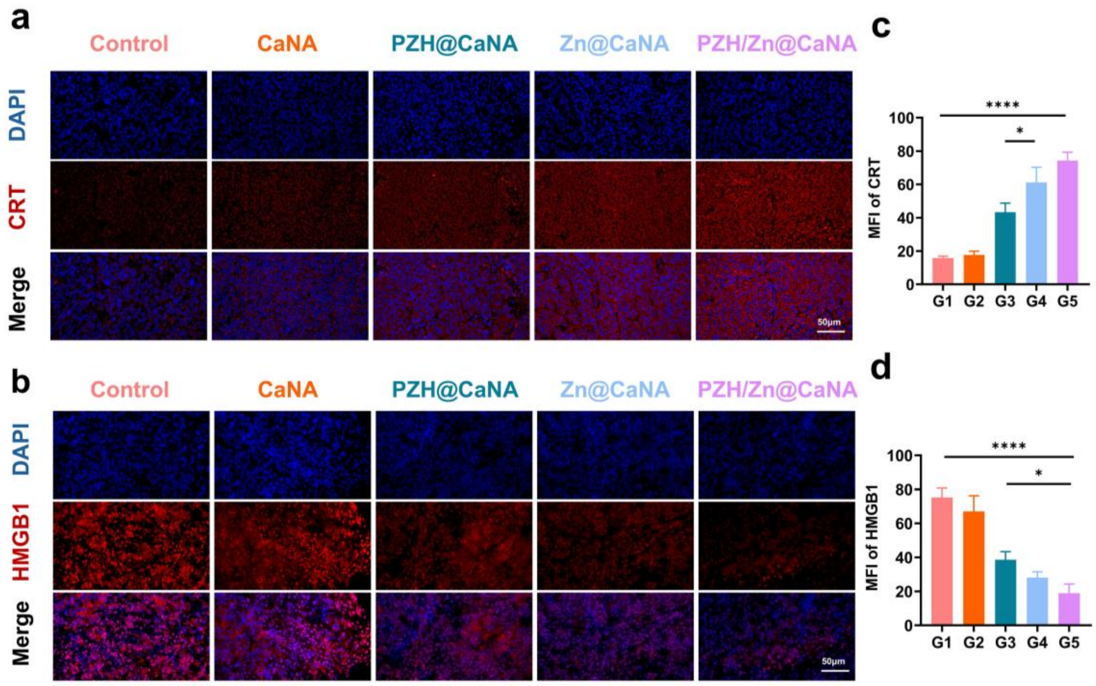


**Figure S23.** (a) Immunofluorescence images of CRT, (b) HMGB1 of tumors in different treatment groups (scar bar: 50 μm). (c) Quantitative statistics of immunofluorescence images of CRT and (d) HMGB1 in different treatment groups. (G1: Control; G2: CaNA; G3: PZH@CaNA; G4: Zn@CaNA; G5: PZH/Zn@CaNA). (*$p < 0.05$, **$p < 0.01$, ***$p < 0.001$, ****$p < 0.0001$).

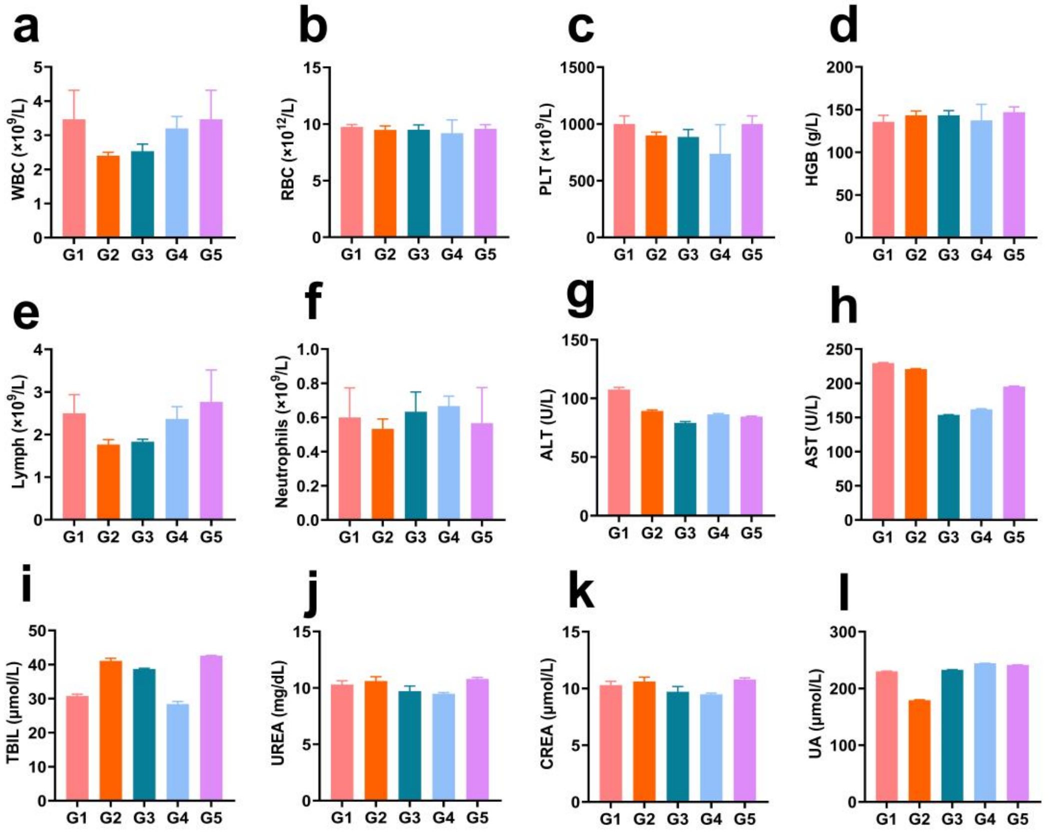


**Figure S24.** Routine blood, liver and kidney function analysis of C57BL/6J mice in different treatment groups. (G1: Control; G2: CaNA; G3: PZH@CaNA; G4: Zn@CaNA; G5: PZH/Zn@CaNA).

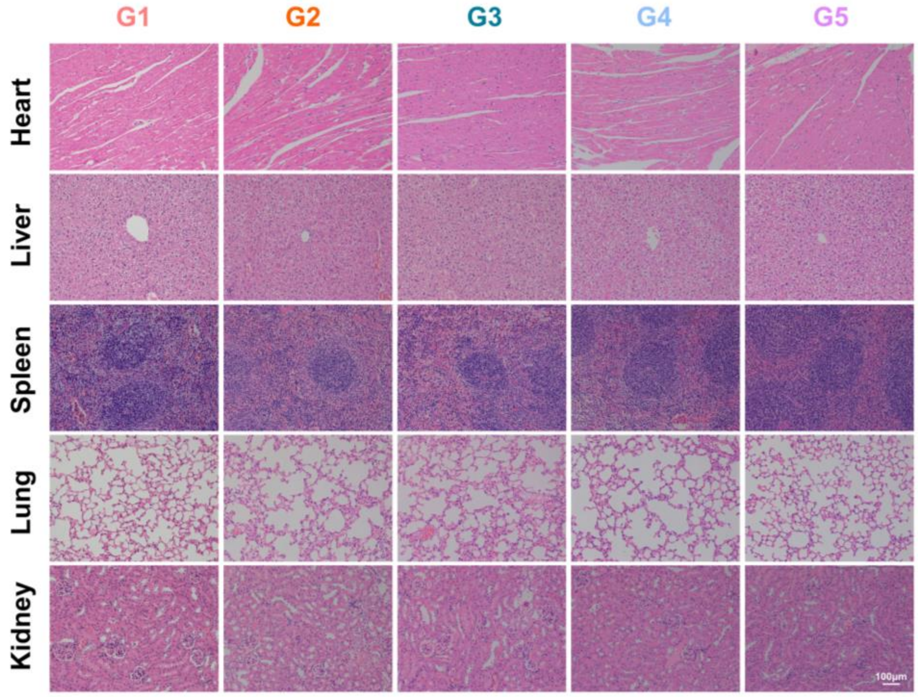


**Figure S25.** H&E staining of heart, liver, spleen, lung and kidney from healthy mice in different treatment groups (scar bar: 100 μm). (G1: Control; G2: CaNA; G3: PZH@CaNA; G4: Zn@CaNA; G5: PZH/Zn@CaNA).

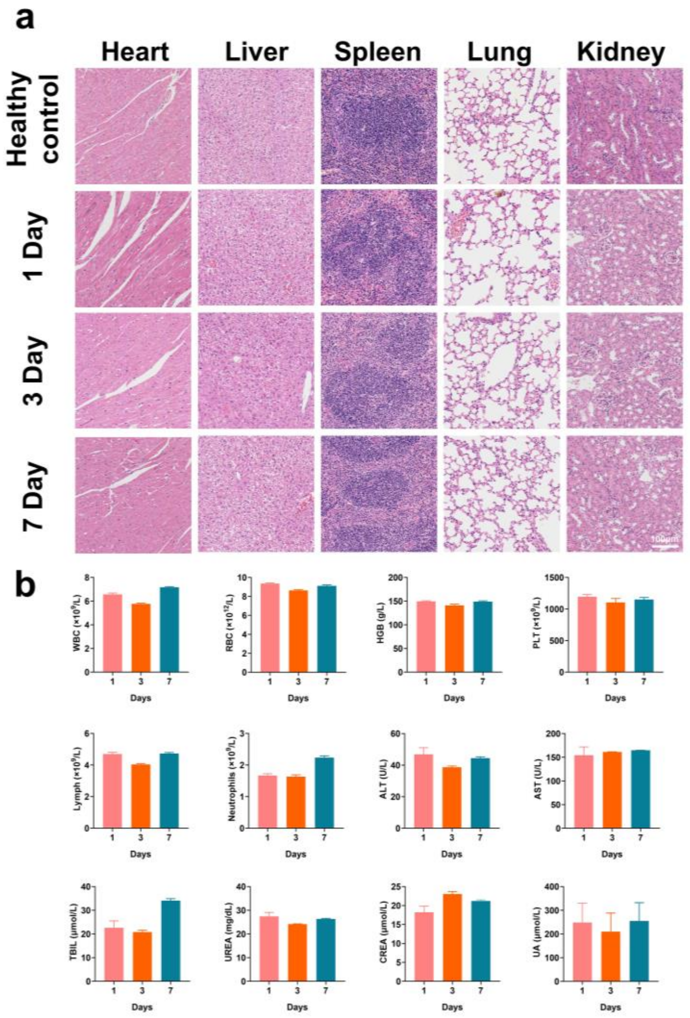


**Figure S26.** (a) H&E optical microscopic images of normal organs after l day, 3 days and 7 days post-injection of PZH/Zn@CaNA. (scar bar: 100 μm). (b) Routine blood, liver and kidney function analysis of C57BL/6J mice after 1 day,3 days and 7 days.

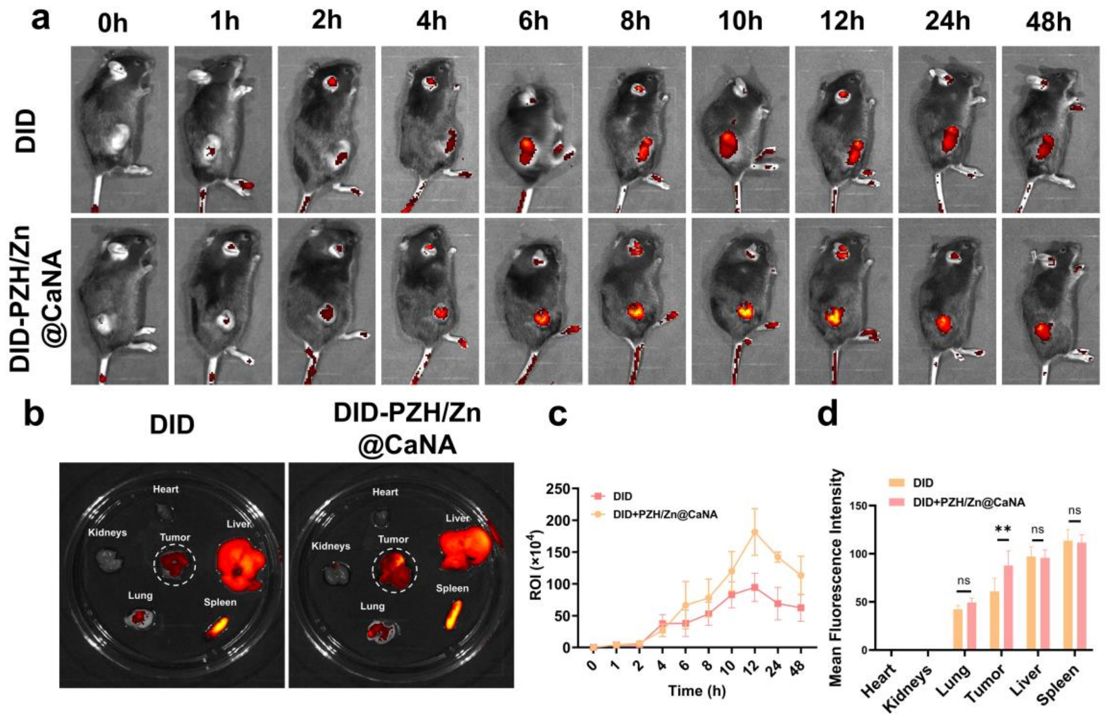


**Figure S27.** (a) Bioluminescence imaging data of drug accumulation in tumor tissues of iMWA mice were collected at different time points after injection of DID-PZH/Zn@CaNA and DID. (b) In vitro fluorescence images of tumor tissues and major organs of iMWA mice were obtained 12 h after injection of DID-PZH/Zn@CaNA and DID dye. (c) The fluorescence intensity of drug accumulation in tumor tissues of iMWA mice was plotted at different time points after injection of DID-PZH/Zn@CaNA and DID dye. (d) Quantitative analysis of fluorescence intensity of tumor tissues and major organs of iMWA mice 12 h after injection of DID-PZH/Zn@CaNA and DID dye. (*$p < 0.05$, **$p < 0.01$, ***$p < 0.001$, ****$p < 0.0001$).

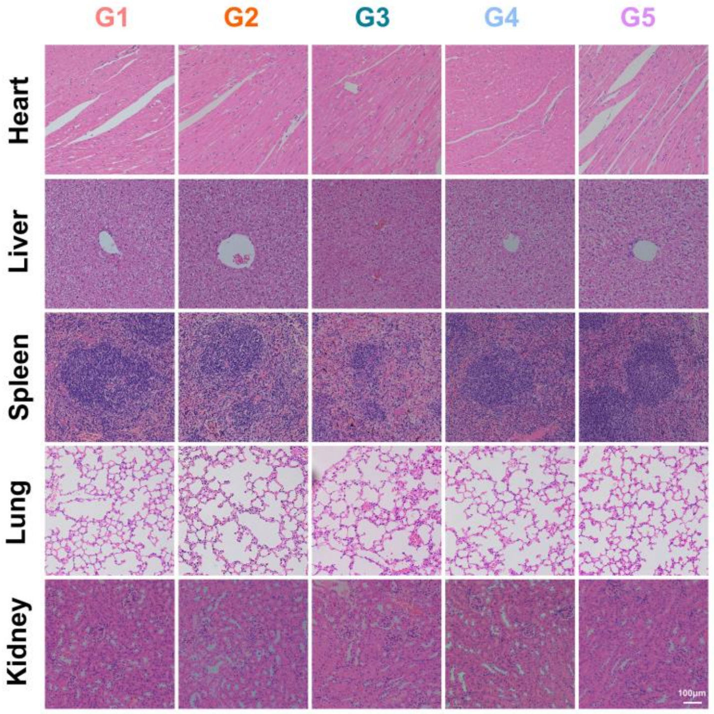


**Figure S28.** H&E staining of heart, liver, spleen, lungs and kidneys of tumor-bearing mice in different treatment groups (scar bar: 100 μm). (G1: Control; G2: CaNA; G3: PZH@CaNA; G4: Zn@CaNA; G5: PZH/Zn@CaNA).

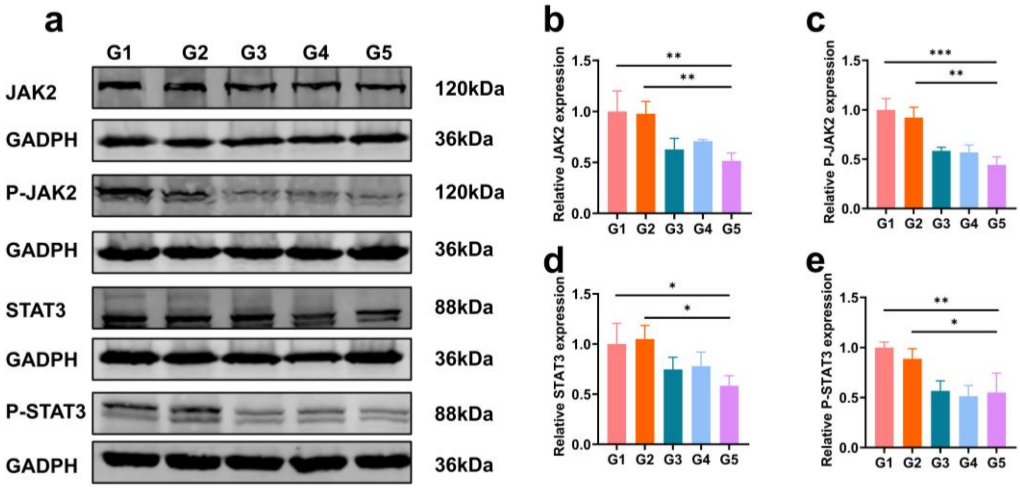


**Figure S29.** (a) WB analysis of protein expression levels associated with the JAK2-STAT3 signaling pathway in tumor with different treatment groups and quantitative analysis of WB images of (b) JAK2, (c) P-JAK2, (d) STAT3, (e) P-STAT3. (G1:

Control; G2: CaNA; G3: PZH@CaNA; G4: Zn@CaNA; G5: PZH/Zn@CaNA). (*p < 0.05, **p < 0.01, ***p < 0.001, ****p < 0.0001).

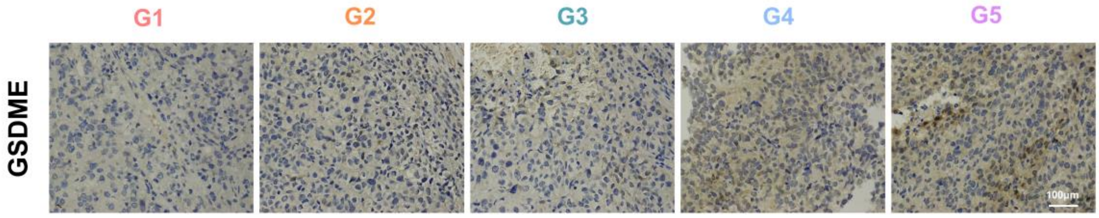


**Figure S30.** Immunohistochemistry of GSDME protein of tumor cells from C57BL/6J mice in different treatment groups (scar bar: 100 μm). (G1: Control; G2: CaNA; G3: PZH@CaNA; G4: Zn@CaNA; G5: PZH/Zn@CaNA).

**References:**

[1] T. Su, M. Huang, J. Liao, S. Lin, P. Yu, J. Yang, Y. Cai, S. Zhu, L. Xu, Z. Peng, S. Peng, S. Chen, M. Kuang, Insufficient Radiofrequency Ablation Promotes Hepatocellular Carcinoma Metastasis Through N6-Methyladenosine mRNA Methylation-Dependent Mechanism [J]. Hepatology. 74(3) (2021) 1339-1356.

[2] G. Qiu, D. Wang, P. Xie, Z. Li, N. Zhou, X. Zhang, X. Wang, J. Tang, J. Cao, J. Liu, Harnessing bimetallic iMWA nanosensitizer to unleash ferroptosis and calcium overload: Unlocking tumor vulnerability for potentiated iMWA therapy against hepatocellular carcinoma [J]. Chem. Eng. J. (2024) 153368.

[3] X. Zhu, T. Li, Q. Wang, K. Yan, S. Ma, Y. Lin, G. Zeng, J. Liu, J. Cao, D. Wang, Dual-Synergistic Nanomodulator Alleviates Exosomal PD-L1 Expression Enabling Exhausted Cytotoxic T Lymphocytes Rejuvenation for Potentiated iRFA-Treated Hepatocellular Carcinoma Immunotherapy [J]. ACS Nano. 18(47) (2024) 32818-32833.

[4] Y. Zhu, Z. Yang, Z. Pan, Y. Hao, C. Wang, Z. Dong, Q. Li, Y. Han, L. Tian, L. Feng, Z. Liu, Metallo-alginate hydrogel can potentiate microwave tumor ablation for synergistic cancer treatment [J]. Sci Adv. 8(31) (2022) eabo5285.

[5] Y. Wu, B. Liu, Y. Yan, C. Gong, K. Wang, N. Liu, Y. Zhu, M. Li, C. Wang, Y. Yang, L. Feng, Z. Liu, Thermal-responsive activation of engineered bacteria to trigger antitumor immunity post microwave ablation therapy [J]. Nat Commun. 15(1) (2024) 10503.

[6] L. Shen, Z. Yang, Y. Zhong, Y. Bi, J. Yu, Q. Lu, Y. Su, X. Chen, Z. Zhao, G. Shu, M. Chen, L. Cheng, L. Feng, C. Lu, Z. Liu, J. Ji, Cholesterol Targeted Catalytic Hydrogel Fueled by Tumor Debris can Enhance Microwave Ablation Therapy and Anti-Tumor Immune Response [J]. Adv Sci (Weinh). (2024) e2406975.

[7] Z. Dong, L. Feng, Y. Hao, M. Chen, M. Gao, Y. Chao, H. Zhao, W. Zhu, J. Liu, C. Liang, Q. Zhang, Z. Liu, Synthesis of Hollow Biomineralized CaCO3-Polydopamine Nanoparticles for Multimodal Imaging-Guided Cancer Photodynamic Therapy with Reduced Skin Photosensitivity, Journal of the American Chemical Society 140(6) (2018) 2165-2178.
[8] B. Xie, L. Dong, L. Wang, R. Wang, C. Li, Supramolecularly engineered bacteria mediated calcium overload and immunotherapy of tumors, Theranostics 14(17) (2024) 6560-6570.
[9] P. Zheng, B. Ding, G. Zhu, C. Li, J. Lin, Biodegradable Ca2+ Nanomodulators Activate Pyroptosis through Mitochondrial Ca2+ Overload for Cancer Immunotherapy, Angew Chem Int Ed Engl 61(36) (2022) e202204904.
